# Classical Swine Fever Virus Envelope Glycoproteins E^rns^, E1, and E2 Activate IL-10-STAT1-MX1/OAS1 Antiviral Pathway via Replacing Classical IFNα/β

**DOI:** 10.3390/biom15020200

**Published:** 2025-01-31

**Authors:** Liyuan Zhang, Dongli Liang, Yu Tian, Jiaxin Liang, Xiaoquan Li, Cheng Liu, Jingjing Liang, Ting Rong Luo, Xiaoning Li

**Affiliations:** 1 State Key Laboratory for Conservation and Utilization of Subtropical Agro-Bioresources, Guangxi University, Nanning 530004, China; 2118402003@st.gxu.edu.cn (L.Z.); 2218302019@st.gxu.edu.cn (D.L.); 2318393068@st.gxu.edu.cn (Y.T.); 2318393042@st.gxu.edu.cn (J.L.); dan1986_7@sr.gxmu.edu.cn (X.L.); liucheng0621@gxu.edu.cn (C.L.); jiliang@upenn.edu (J.L.); 2 College of Animal Sciences and Veterinary Medicine, Guangxi University, Nanning 530004, China; 3 Guangxi Zhuang Autonomous Region Engineering Research Center of Veterinary Biologics, Nanning 530004, China; 4 Guangxi Key Laboratory of Animal Breeding, Disease Control and Prevention, Nanning 530004, China

**Keywords:** CSFV, surface glycoproteins, STAT1, interleukin-10, MX1, OAS1

## Abstract

Classical swine fever (CSF) is an acute and often fatal disease caused by CSF virus (CSFV) infection. In the present study, we investigated the transcriptional profiles of peripheral blood mononuclear cells (PBMCs) in pigs infected with CSFV. The results revealed that CSFV inhibits IFNα/β production, but up-regulates the expression of signal transducer and activator of transcription 1 (STAT1); this result was verified in vitro. Interestingly, STAT1 is typically a downstream target of IFNα/β, raising the question of how CSFV can inhibit IFNα/β expression, yet up-regulate STAT1 expression. To explore this further, we observed that UV-treated CSFV induced STAT1 expression. Our results demonstrated that CSFV E^rns^, E1, and E2 could up-regulate STAT1 expression within the host cell cytoplasm and facilitate its translocation into the nucleus. The E^rns^, E1, and E2 proteins also separately induced the up-regulation of interleukin (IL)-10; IL-10 acts as the communicator connecting E^rns^, E1, and E2 proteins to STAT1, leading to the subsequent up-regulation, phosphorylation, and nuclear translocation of STAT1. Silencing of IL-10 down-regulated STAT1 expression. Finally, MX1 and OAS1 were identified as downstream targets of the IL-10-STAT1 pathway. In summary, a novel IL-10-STAT1 pathway independent of IFNα/β induced by CSFV E^rns^, E1, and E2 was identified in this study.

## 1. Introduction

Classical swine fever (CSF) is primarily characterized by immunosuppression, reproductive disorders, a reduction in platelet mass, and diffuse intravascular coagulation [1]. This disease results in substantial economic losses for the swine industry in endemic regions worldwide, especially China [2,3,4]. According to data from the Ministry of Agriculture, the southern provinces of China—specifically Yunnan, Guangdong, Fujian, and Guangxi—are among the most severely impacted by CSF virus (CSFV) outbreaks [5,6,7]. CSFV typically encodes a large polyprotein derived from an open reading frame (ORF), which is subsequently cleaved into four structural proteins: Core (C), E^rns^, E1, and E2. In addition, the virus produces eight nonstructural proteins following digestion by viral or host proteases [8]. The conserved C protein protects the internal viral genome to ensure viral transcription and replication. The S243-A255 sequence of the C protein is highly conserved; the deletion of this sequence results in a loss of the function of the C protein in viral infection. CSFV replication can be inhibited in vitro by capsid-targeted viral inactivation (CTVI) [9,10,11,12,13,14]. The glycoprotein E^rns^, also known as E0, has ribonucleolytic activity and attaches to heparan sulfate to enter and induce apoptosis of susceptible host immune cells [15,16]. In addition, E^rns^ can bind to double-stranded RNA (dsRNA) and antagonize the expression of interferon (IFN)β [17,18]. E1 has three glycosylation sites that alter the virulence of CSFV [19,20]. The glycoprotein E2 is an essential component of the virus surface and acts as an antigen that induces protective immunity of the host. The E2 protein is primarily responsible for viral uptake into susceptible cells, immune invasion, and virulence exertion during CSFV infection. E2 plays a critical role in determining the virulence of pigs infected with CSFV. Furthermore, mutations in the C-terminal region of CSFV E2 have been shown to reduce the virus’s virulence [21,22,23]. E1 and E2 form a heterodimer through disulfide bonds that mediates the entry of CSFV into the host cell [24].

CSFV infection has been shown to activate several innate immune signaling pathways, including the RIG-I/MAVS, ROS/RLR, AMPK/mTOR, and JAK/STAT signaling pathways [25,26,27,28,29,30,31,32,33]. Among these, STAT1 is a key component of the JAK/STAT pathway, functioning as a downstream target of IFNα/β and playing a critical role in the activation of various antiviral proteins. STAT1 exists in two transcription isoforms (STAT1-α and STAT1-β). STAT1-α possesses a complete transcriptional activation domain, with phosphorylation sites at positions 701 and 727 serving as the primary sites responsible for activating signal transduction [34]. In fact, interferon does not directly inactivate the virus; rather, it synthesizes antiviral proteins that inhibit viral replication. The interaction of CSFV N_pro_ and IRF3 inhibits IFNα/β production [35], and the binding of E^rns^ to dsRNA inhibits IFNβ activation [18].

Our research group discovered that CSFV infection could inhibit the transcription of immune response genes, including IFNα/β [36]. Since IFNα/β is responsible for the activation of STAT1, CSFV infection should inhibit STAT1 expression. Our studies indicated that CSFV infection suppressed IFNα/β production but up-regulated STAT1 expression, suggesting that CSFV might escape the innate immune response by inhibiting the expression of IFNα/β. Alternatively, the host cell might activate STAT1 rather than IFNα/β to protect against viral infection.

This study aimed to explore the mechanism by which the host utilizes the STAT1 pathway to counteract CSFV infection and achieve immune protection.

## 2. Materials and Methods

### 2.1. Cells

The STAT1 gene knockout PK-15 (PK-15^STAT1-/-^) cell line was constructed in our laboratory [37]. PK-15 cells and human embryonic kidney (HEK) 293T cells were acquired from Procell Co., Ltd. (Wuhan, China). All cell lines were cultured in Dulbecco’s modified Eagle’s medium (DMEM) supplemented with 10% fetal bovine serum (FBS).

### 2.2. Viruses and Plasmids

In this study, we employed the standard virulent CSFV Shimen strain. The five recombinant eukaryotic expression vectors—pCMV-Core^-HA^, pCMV-E^rns-His^, pCMV-E1^-Myc^, and pCMV-E2^-Flag^, and pcDNA3.0-STAT1^-His^—were constructed in our laboratory.

### 2.3. Transfection/Infection Assays

PK-15 cells were initially cultured in 12-well plates and transfected with either the pCMV plasmid or recombinant eukaryotic expression plasmids, including pCMV-Core^-HA^, pCMV-E^rns-His^, pCMV-E1^-MYC^, and pCMV-E2^-Flag^ (1.0 µg), for a duration of 6 h. Additionally, two control groups were established: one consisting of PK-15 cells infected with CSFV at a multiplicity of infection (MOI) of 1, and another comprising PK-15 cells incubated with 100 IU/mL IFNα. Cell extracts were collected at 12, 24, 48, and 72 h. Specific antibodies were employed for Western blot analysis to detect both cellular and viral proteins. Total RNA was extracted from the infected cells using the standard TRIzol (Thermo Fisher Scientific, Cambridge, MA, USA) RNA extraction protocol for subsequent quantitative RT-PCR analysis.

### 2.4. ELISA

ELISA kits were utilized for the detection of porcine interleukin (IL)-10 (Shanghai ZCIBIO Technology Co., Ltd., Shanghai, China) as well as the CSFV E^rns^, E1, and E2 proteins (Shanghai Yuanji Chemical Co., Ltd., Shanghai, China). Initially, PK-15 cells were transfected with constructs encoding the following (tagged) CSFV proteins: pCMV-E^rns-His^, pCMV-E1^-Myc^, and pCMV-E2^-flag^. Subsequently, the expression of IL-10 and the CSFV E^rns^, E1, and E2 proteins were quantified using commercially available ELISA kits, following the manufacturer’s protocol.

### 2.5. Western Blot (WB) Analysis

Total proteins were isolated from the cells, and the target proteins were separated and subsequently transferred onto polyvinylidene fluoride (PVDF) membranes. The membranes were blocked with 5% skimmed milk overnight at 4 °C. Following blocking, the membranes were probed at 37 °C for 60 min with primary antibodies (Abs) against STAT1 (10144-2-AP), Myc tag (16286-1-AP), lamin B1 (12987-1-AP), OAS1 (14955-1-AP), MX1 (13750-1-AP), His tag (10001-0-AP), and Flag tag (20543-1-AP) (all from Proteintech Co., Ltd., Wuhan, China); phosphorylated (p) STAT1 (9167S), IFNα (6B18), and IFNβ (D2J1D) (from Cell Signaling Technology, Inc., Danvers, MA, USA); CSFV E1/E2 (9011) (from JENO Biotech, Seoul, Korea); CSFV E2 (orb500382) (from Biorbyt Ltd., Cambridge, England); and β-actin (CW0096A) (from Beijing CoWin Biotech Co., Ltd., Beijing, China). After incubation with the primary antibodies, the membranes were washed three times with 1× TBST, and then probed at 37 °C for 60 min with the appropriate secondary antibodies. A color prestained protein marker (10–250 kDa, Dual colors) (P8028S) was obtained from UElandy Co., Ltd. (Beijing, China) and used for molecular weight reference. The target protein bands were visualized using a BCIP/NBT staining kit (Beyotime Co., Ltd., Beijing, China) and quantified using ImageJ (version 2.0) software (https://imagej.net/ij/) (accessed on 1 March 2024).

### 2.6. RT-qPCR

Cellular RNA was isolated using the RNA Isolation Kit (Tiangen Biotech Co., Ltd., Beijing, China) following the manufacturer’s instructions. The relative mRNA expression of STAT1, CSFV C, E^rns^, E1, E2, TGFβ1, TGFβ3, IL-8, IL-10, IFNα, IFNβ, IFNγ, CC motif chemokine receptor (CCR)2, CCR3, OAS1, MX1, ISG20, and GAPDH were evaluated using a LightCycler 96 PCR detection system (Roche Diagnostics, Shanghai, China). The gene-specific primers used in this study are listed in Table S1. The relative expression was determined using the 2^−ΔΔCt^ method, with *GAPDH* serving as the internal reference gene for normalization.

### 2.7. Inactivation of CSFV

A non-infectious form of CSFV was prepared using the following procedure. Briefly, a CSFV solution (100 μL) was exposed to ultraviolet (UV) light for 35 min. The UV-treated CSFV solution was confirmed to be inactivated and was subsequently used to infect PK-15 cells. Total protein and RNA samples were then collected at 48 h post-inoculation (hpi) for immunofluorescence (IF) and RT-qPCR analyses, respectively.

### 2.8. siRNA Assay

The cells were transfected with siRNA using the Invitrogen™ Oligofectamine™ reagent (Fisher Scientific Company, Ottawa, ON, Canada). The siRNA sequences designed for silencing IL-10 and the control siRNA were generated using the following primer sequences: for IL-10, 5′-GCUCAGCACUGCUCUAUUGTT-3′ (sense) and 5′-CAAUAGAGCAGUGCUGAGCTT-3′ (antisense); for the control, 5′-UUCUCCGAACGUGUCACGU-3′ (sense) and 5′-ACGUGACACGUUCGGAGAA-3′ (antisense).

### 2.9. IFA

The PK-15 cells were subjected to various treatment protocols. At 48 h, the treated cells were fixed using a 1:1 mixture of methanol and acetone, and then incubated with a specific primary antibody. Following this, the cells were stained with a corresponding secondary antibody. Finally, the cells were visualized and imaged using a fluorescence microscope.

### 2.10. Nuclear Protein and Cytoplasmic Protein Extraction

PK-15 cells were transfected with pCMV-E^rns-His^, pCMV-E1^-Myc^, and pCMV-E2^-flag^, respectively. Following transfection, cytoplasmic and nuclear proteins were isolated using a Nuclear and Cytoplasmic Protein Extraction Kit (Beyotime Institute of Biotechnology) (Shanghai, China) according to the manufacturer’s instructions.

### 2.11. IL-10 Treatment

Recombinant porcine IFNα and IL-10 were purchased from Intel Phil Co., Ltd. (Hefei, China). The initial concentrations of porcine IFNα and IL-10 were 10,000 IU/mL. Consequently, these concentrations were adjusted to 100 IU/mL in the experiments.

### 2.12. Statistical Analysis

The data are presented as mean ± standard deviation ($\overset{-}{x}$ ± s.d). Statistical analyses were conducted using GraphPad Prism (version 8.0; MA, USA). Comparisons between two groups were conducted using Student’s *t*-test, while the Tukey multiple comparison test was applied for analyses involving three or more groups. A probability (*p*) value of less than 0.05 was considered statistically significant.

## 3. Results

### 3.1. IFNα/β Down-Regulation and STAT1 Up-Regulation in Response to CSFV

To characterize changes in the innate immune response of pigs due to CSFV infection, the pigs were infected with a standard virulent CSFV strain, Shimen; PMBCs were then isolated from the infected pigs [36]. The fold changes in the mRNA levels of the selected genes were analyzed at 1, 3, 6, and 9 dpi using a microarray (Table 1). The levels of both IFNα and IFNβ mRNA showed no obvious changes in response to CSFV infection (<2.0 folds).

**Figure 1:**
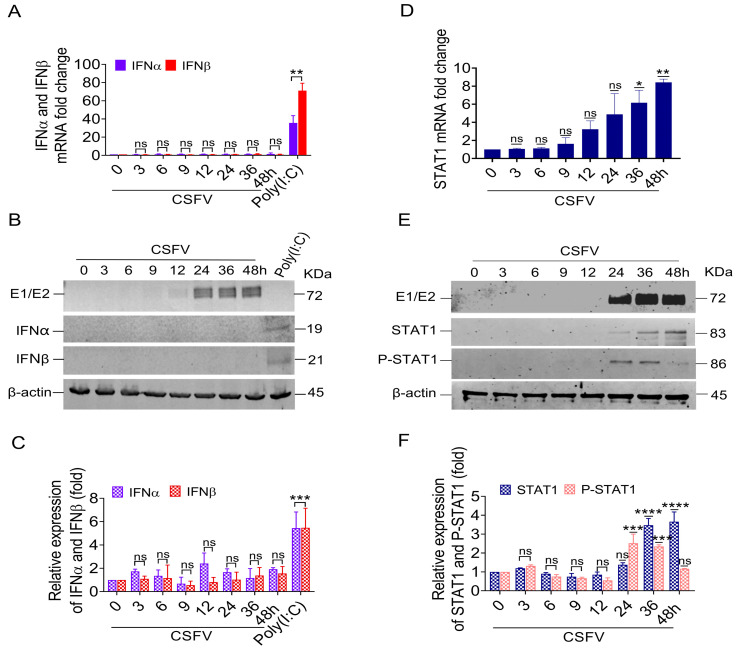
IFNα/β down-regulation and STAT1 up-regulation in response to CSFV. (**A**,**B**) The mRNA levels and protein expression of IFNα and IFNβ were analyzed in PK-15 cells infected with CSFV (MOI = 1) or transfected with 50 μg poly(I:C) by RT-qPCR (**A**) and WB (**B**) analysis. (**D**) The mRNA levels of STAT1 were analyzed in PK-15 cells infected with the CSFV (MOI = 1) at 0, 3, 6, 9, 12, 24, 36, and 48 hpi by RT-qPCR analysis. (**E**) The protein levels of E1/E2, STAT1, and P-STAT1 (Tyr 701) were analyzed by WB analysis. (**C**,**F**) The relative expression values of IFNα, IFNβ, STAT1, and P-STAT1 proteins were quantified using Image J software (* *p* < 0.05, ** *p* < 0.01, *** *p* < 0.001, **** *p* < 0.0001). Original images of the western blots can be found at Supplementary Materials.

To verify the reliability of the microarray data, we measured the mRNA levels and protein expression of IFNα/β genes in PK-15 cells that were infected in vitro with 1 MOI of CSFV. The results showed no obvious change to IFNα/β mRNA expression following CSFV infection. In contrast, IFNα/β mRNA expression was induced by Poly(I:C) (Figure 1A). We did not detect either IFNα or IFNβ protein via Western blotting of cell lysates from CSFV-infected PK-15 cells, while the PK-15 cells expressed both IFN protein isoforms upon Poly(I:C) treatment (Figure 1B,C).

Simultaneously, the mRNA levels of the STAT1 gene in the PBMC of pigs infected with CSFV were gradually increased; their changed folds at 1, 3, 6, and 9 dpi were 1.26, 1.44, 1.81, and 2.07, respectively (Table 1). Similarly, the mRNA level of the STAT1 gene increased in CSFV-infected PK-15 cells in vitro from 36 to 48 hpi compared with mock cells (Figure 1D). At these time points, we also detected STAT1 and phosphorylated STAT1 at the protein level following CSFV E1/E2 expression via Western blot. The results showed that STAT1 protein levels were up-regulated by 3.45-fold at 36 hpi and 3.6-fold at 48 hpi (Figure 1E,F), consistent with the E1/E2 protein expression levels. The phosphorylation of STAT1 at Y701 (P-STAT1^Y701^) was up-regulated by 2.45-fold at 24 hpi and 2.3-fold at 36 hpi (Figure 1E,F).

These findings revealed that the up-regulation of STAT1 and P-STAT1^Y701^ protein levels was related to the protein expression of E1/E2, indicating that CSFV-induced STAT1 up-regulation might be independent of IFNα/β.

### 3.2. STAT1 Expression Was Up-Regulated by UV-Treated CSFV In Vitro

**Figure 2:**
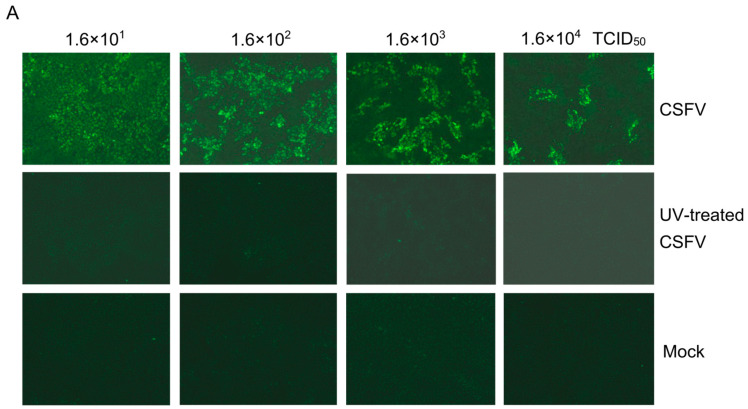

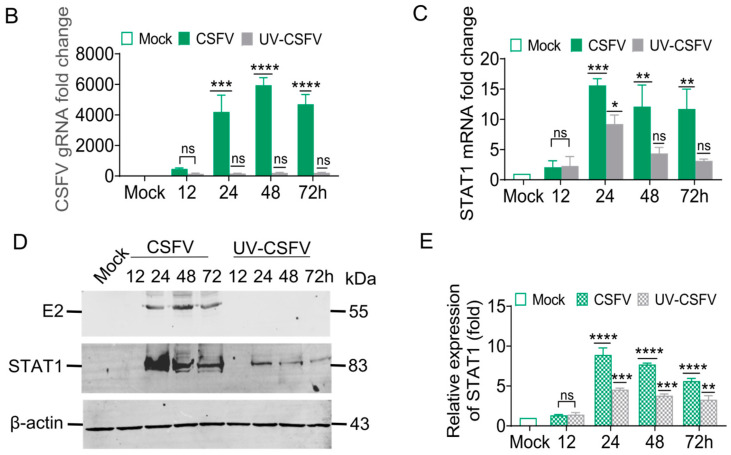
STAT1 expression was up-regulated by UV-treated CSFV in vitro. (**A**) PK-15 cells were inoculated with CSFV and UV-treated CSFV at 10^1^, 10^2^, 10^3^, and 10^4^ TCID_50_. CSFV replication was assessed using an anti-CSFV E2 Ab at 48 hpi by IF analysis. CSFV gRNA (**B**) and STAT1 mRNA (**C**) levels in PK-15 cells inoculated with CSFV and UV-treated CSFV at 12, 24, 48, and 72 hpi were determined by RT-qPCR analysis. (**D**) CSFV E2 and STAT1 protein levels at 12, 24, 48, and 72 hpi were measured by WB analysis. (**E**) The relative expression values of STAT1 protein were quantified using Image J software (* *p* < 0.05, ** *p* < 0.01, *** *p* < 0.001, **** *p* < 0.0001). Original images of the western blots can be found at Supplementary Materials.

CSFV infection up-regulated STAT1 expression via an independent IFNα/β pathway. To identify which component of CSFV is responsible for STAT1 up-regulation, a non-infectious CSFV was prepared by exposure to UV light for 35 min (UV-CSFV). The PK-15 cells were inoculated with a series of dilutions (10^−1^, 10^−2^, 10^−3^, 10^−4^ TCID_50_) of CSFV and the status of viral replication was detected by IFA; they exhibited green fluorescence, indicating the replication of CSFV (Figure 2A). However, the PK-15 cells inoculated with UV-CSFV did not emit green fluorescence, indicating a lack of CSFV replication (Figure 2A). The PK-15 cells inoculated with UV-CSFV showed no viral replication from 24 to 72 hpi (Figure 2B,D). These data indicate that CSFV, but not UV-CSFV, replicated in PK-15 cells in vitro.

The mRNA and protein levels of STAT1 in PK-15 cells inoculated with UV-CSFV or CSFV were analyzed, respectively. The STAT1 mRNA levels were increased in PK-15 cells inoculated with both UV-CSFV and CSFV. However, CSFV induced higher mRNA levels of STAT1 than UV-CSFV (Figure 2C). STAT1 protein levels were relatively high in PK-15 cells inoculated with UV-CSFV or CSFV. Similarly, CSFV infection induced higher protein levels of STAT1 than UV-CSFV (Figure 2D,E).

The above results substantiate that the UV-treated CSFV induces the mRNA and protein expression of STAT1, suggesting a potential association between STAT1 expression and the outer structural proteins of CSFV, such as the Core, E^rns^, E1, and E2 proteins.

### 3.3. CSFV Up-Regulated STAT1 Expression via E^rns^, E1, and E2, but Not the Core Protein

To identify the CSFV structural proteins that promote STAT1 expression, the mRNA and protein levels of STAT1 in PK-15 cells separately transfected with pCMV-Core^-HA^, pCMV-E^rns-His^, pCMV-E1^-Myc^, and pCMV-E2^-Flag^ were measured.

**Figure 3:**
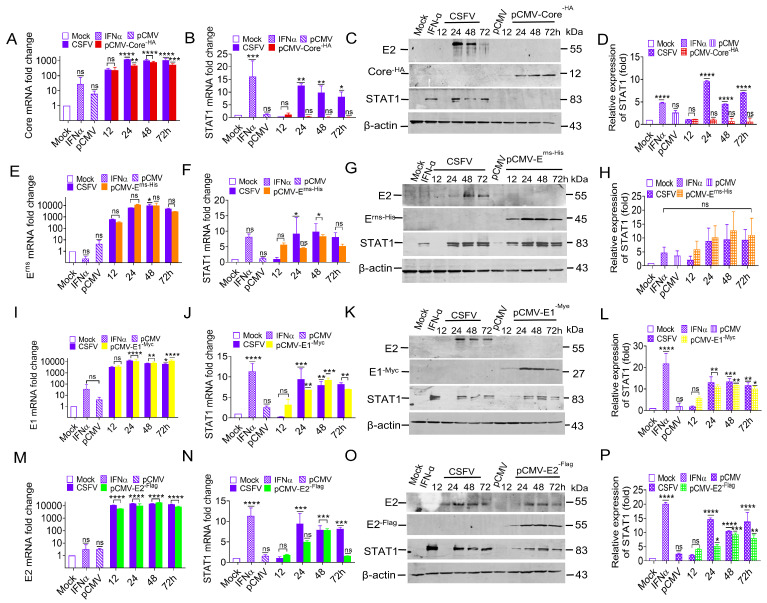
CSFV up-regulated STAT1 expression via E^rns^, E1, and E2, but not the Core protein. PK-15 cells were infected with CSFV (MOI = 1) and separately transfected with 1.0 μg of the recombinant eukaryotic expression vectors pCMV-Core^-HA^ (**A**–**D**), pCMV-E^rns-His^ (**E**–**H**), pCMV-E1^-Myc^ (**I**–**L**), and pCMV-E2^-Flag^ (**M**–**P**). PK-15 cells incubated with 100 IU/mL of IFNα served as the positive control, while PK-15 cells transfected with an empty pCMV vector were utilized as the negative control. Samples were collected 72 h later for subsequent analysis. CSFV Core (**A**), E^rns^ (**E**), E1 (**I**), E2 (**M**), and STAT1 (**B**,**F**,**J**,**N**) mRNA levels were measured by RT-qPCR analysis at 12, 24, 48, and 72 h. (**C**,**G**,**K**,**O**) CSFV E2, Core^-HA^, E^rns-His^, E1^-Myc^, E2^-Flag^, and STAT1 protein levels at 12, 24, 48, and 72 h were measured by WB analysis. (**D**,**H**,**L**,**P**) The relative expression values of STAT1 protein were quantified using Image J software (* *p* < 0.05, ** *p* < 0.01, *** *p* < 0.001, **** *p* < 0.0001). Original images of the western blots can be found at Supplementary Materials.

To understand if the Core protein is also involved in STAT1 up-regulation, the Core protein was also expressed in PK-15 cells with either CSFV infection or transfection with the pCMV-Core^-HA^ construct. The mRNA levels of the Core were increased in both the transfection group and virus infection group (Figure 3A). However, STAT1 mRNA and protein changes were undetectable in PK-15 cells transfected with pCMV-Core^-HA^ at 12 to 72 h post-transfection (hpt) (Figure 3B–D). These results indicate that the CSFV Core protein does not regulate STAT1 expression.

To confirm if the E^rns^ protein regulates STAT1 expression or not, the PK-15 cells were used to express the E^rns^ protein by infecting CSFV or transfecting with pCMV-E^rns-His^. The E^rns^ mRNA levels were increased in both the transfection group and virus infection group (Figure 3E). Simultaneously, the levels of STAT1 mRNA and protein exhibited a significant increase, which was consistent with the expression trend of the E^rns^ protein observed in both the transfection group and virus infection group (Figure 3F–H). These results suggest that the CSFV E^rns^ protein might be involved with the regulation of STAT1 expression.

To confirm if the E1 protein regulates STAT1 expression or not, the PK-15 cells were used to express E1 by infecting CSFV or transfecting with pCMV-E1^-Myc^. The E1 mRNA levels were increased in both the transfection group and virus infection group (Figure 3I). Moreover, the STAT1 mRNA and protein levels were significantly increased in both the transfection group and virus infection group (Figure 3J–L). These results demonstrate that the CSFV E1 protein also participated in the regulation of STAT1 expression.

To confirm if the E2 protein regulates STAT1 expression or not, the PK-15 cells were used to express E2 by infecting CSFV or transfecting with pCMV-E2^-Flag^. The E2 mRNA levels were up-regulated in both the transfection group and virus infection group (Figure 3M). Moreover, STAT1 mRNA and protein levels were up-regulated in the transfection group and virus infection group (Figure 3N–P). These results demonstrate that the CSFV E2 protein participated in the regulation of STAT1 expression.

Collectively, these findings suggest that CSFV up-regulated STAT1 expression via the glycoproteins E^rns^, E1, and E2, but not the Core protein.

### 3.4. STAT1 Translocation to the Nucleus Induced by CSFV E^rns^, E1, and E2

**Figure 4:**
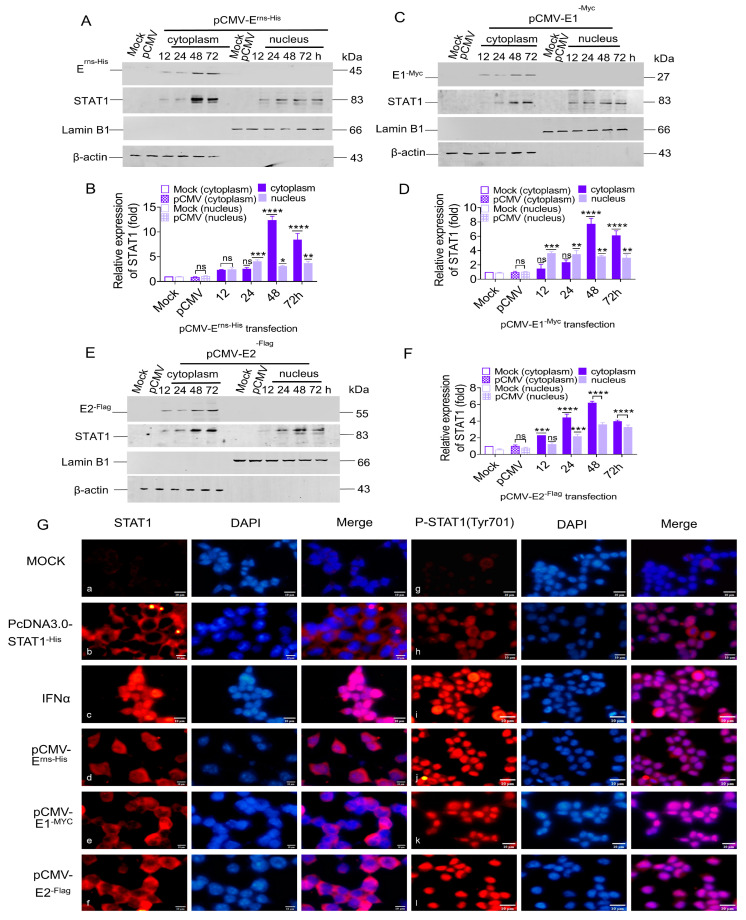
STAT1 translocation to the nucleus induced by CSFV E^rns^, E1, and E2. PK-15 cells were used to express E^rns^, E1, and E2 proteins by transfecting with the pCMV-E^rns-His^, pCMV-E1^-Myc^, and pCMV-E2^-Flag^ vectors, respectively. (**A**,**C**,**E**) The protein levels of STAT1 in the cytoplasm and nucleus of PK-15 cells were measured by WB analysis. (**B**,**D**,**F**) The relative expression values of STAT1 protein were quantified using Image J software. (**G**) HEK 293T cells were separately transfected with the pcDNA3.0-STAT1^-His^, pCMV-E^rns-His^, pCMV-E1^-Myc^, and pCMV-E2^-Flag^ vectors, and treated with IFNα (100 IU/mL). The expression of STAT1 (a–f) and P-STAT1 (g–l) proteins were evaluated at 48 h using anti-STAT1 and anti-P-STAT1 antibodies by IFA analysis (* *p* < 0.05, ** *p* < 0.01, *** *p* < 0.001, **** *p* < 0.0001). Original images of the western blots can be found at Supplementary Materials.

STAT1 can be transferred from the cytoplasm to the nucleus to regulate transcription in the nucleus [38]. To explore the effect of CSFV glycoproteins on STAT1 nuclear transfer, PK-15 cells were used to express E^rns^, E1, and E2 proteins by transfecting with pCMV-E^rns-His^, pCMV-E1^-Myc^, and pCMV-E2^-Flag^, respectively. The cytoplasmic and nuclear proteins were separated from the total cellular proteins. The results showed that the E^rns^ protein up-regulated STAT1 protein expression by 2.3-, 2.5-, 12.3-, and 8.4-fold in the cytoplasm at 12, 24, 48, and 72 hpt, respectively, and by 2.3-, 4.0-, 3.1-, and 3.6-fold in the nucleus (Figure 4A,B). Moreover, E1 protein increased STAT1 protein expression by 1.4-, 2.3-, 7.6-, and 6.0-fold in the cytoplasm at 12, 24, 48, and 72 hpt, respectively, and by 3.6-, 3.4-, 3.1-, and 2.9-fold in the nucleus (Figure 4C,D). Similarly, E2 protein up-regulated STAT1 protein expression by 2.2-, 4.4-, 6.1-, and 3.9-fold in the cytoplasm at 12, 24, 48, and 72 hpt, respectively, and by 1.2-, 2.1-, 3.5-, and 3.2-fold in the nucleus (Figure 4E,F).

To verify if the CSFV E^rns^, E1, and E2 proteins promoted the phosphorylation and nuclear translocation of STAT1, the subcellular localization of STAT1 was examined in HEK 293T cells separately transfected with pCMV-E^rns-His^, pCMV-E1^-Myc^, and pCMV-E2^-Flag^ by IFA. The specific red fluorescence of the STAT1 and P-STAT1 proteins was not observed in mock cells (Figure 4G: a and g), but was present in the cytoplasm and in small amounts in the nucleus of HEK 293T cells transfected with pcDNA3.0-STAT1^-His^ (Figure 4G: b and h). In IFNα-treated cells, the specific red fluorescence of STAT1 was observed in the cytoplasm and nucleus (Figure 4G: c), while P-STAT1 was mainly found in the nucleus (Figure 4G: i). Similarly, the specific red fluorescence of STAT1 was found in the cytoplasm and nucleus of HEK 293T cells individually transfected with pCMV-E^rns-His^, pCMV-E1^-Myc^, and pCMV-E2^-Flag^, while P-STAT1 was only observed in the nucleus (Figure 4G: d, e, f, j, k, and l), indicating that E^rns^, E1, and E2 induced phosphorylation and nuclear translocation of STAT1.

These data demonstrate that the STAT1 could be induced and phosphorylated in the cytoplasm, and then transferred to the nucleus in response to expression of the single protein E^rns^, E1, or E2.

### 3.5. Identification of Genes Upstream of the STAT1 Pathway During CSFV Infection

Previous studies confirmed that the CSFV glycoproteins induced STAT1 production. However, the mechanisms of the CSFV E^rns^, E1, and E2 proteins used to induce STAT1 expression remain unclear. Therefore, factors enabling the communication between CSFV and STAT1 were investigated by screening regulatory genes upstream of the STAT1 pathway during CSFV infection.

**Figure 5:**
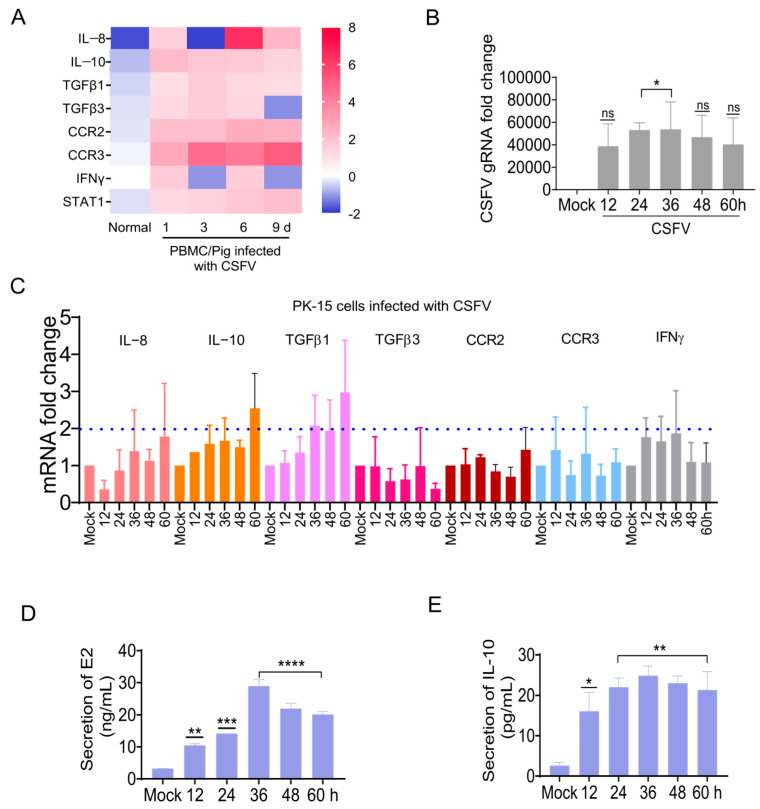
Identification of genes upstream of the STAT1 pathway during CSFV infection. (**A**) A heatmap of changes to the transcriptional levels of IL-8, IL-10, TGFβ1, TGFβ3 CCR2, CCR3, IFNγ, and STAT1 in PBMCs of piglets infected with CSFV. (**B**) PK-15 cells were infected with CSFV (MOI = 1) and the CSFV gRNA copy number was measured by RT-qPCR analysis. (**C**) The transcriptional levels of porcine IL-8, IL-10, TGFβ1, TGFβ3 CCR2, CCR3, and IFNγ at 12, 24, 48, and 60 hpi were measured by RT-qPCR analysis. (D, E) The relative protein expression of CSFV E2 (**D**) and IL-10 (**E**) were measured by ELISA (* *p* < 0.05, ** *p* < 0.01, *** *p* < 0.001, **** *p* < 0.0001).

Microarrays were used to clarify the gene transcription profiles in PBMCs at 1, 3, 6, and 9 dpi for CSFV infection [36]. The IL-8, IL-10, TGFβ1, TGFβ3, CCR2, CCR3, and IFNγ were selected as candidate genes upstream of the STAT1 pathway (Figure 5A).

The mRNA changes in these seven candidate factors were measured in CSFV-infected PK-15 cells by RT-qPCR. The changes in candidate genes we saw via microarray in CSFV-infected pigs were also detected in CSFV-infected PK-15 cells (Figure 5B,C), of which the expression levels of IL-10 and TGFβ1 were increased by more than 2-fold (Figure 5C). To further clarify the effect of CSFV infection, IL-10 protein levels in CSFV-infected PK-15 cells were measured by ELISA. The CSFV E2 protein expression was up-regulated in CSFV-infected cells in vitro (Figure 5D), while the IL-10 protein expression was up-regulated (Figure 5E). These findings confirm that CSFV regulated the expression of some genes upstream of the STAT1 pathway. In particular, IL-10 expression was up-regulated in the PBMCs of piglets infected with CSFV in vivo, as well as CSFV-infected PK-15 cells in vitro.

### 3.6. E^rns^, E1, and E2 Separately Induced IL-10 Up-Regulation In Vitro

**Figure 6:**
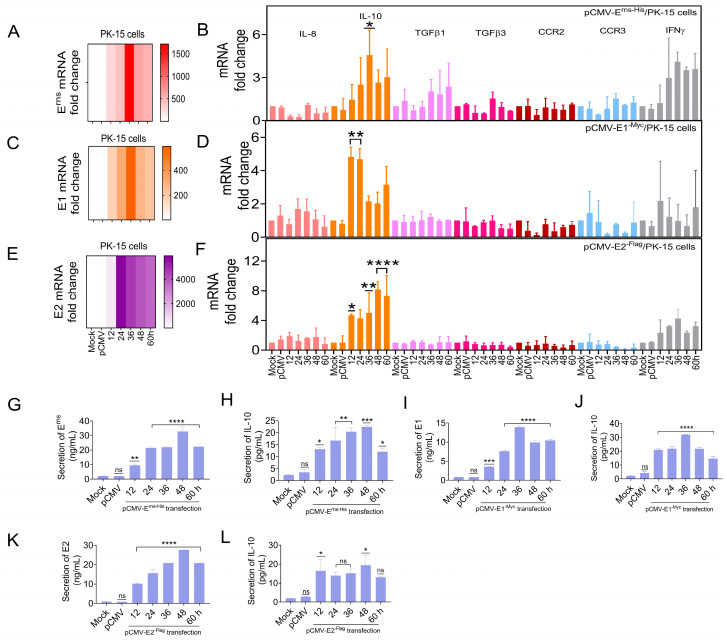
E^rns^, E1, and E2 separately induced IL-10 up-regulation in vitro. PK-15 cells were separately transfected with the pCMV-E^rns-His^, pCMV-E1^-Myc^, and pCMV-E2^-Flag^ vectors (1.0 μg). (**A**–**F**) The mRNA levels of viral E^rns^ (**A**), E1 (**C**), and E2 (**E**), and porcine IL-8, IL-10, TGFβ1, TGFβ3, CCR2, CCR3, and IFNγ in PK-15 cells (**B**,**D**,**F**) at 12, 24, 36, and 48 hpt were measured by RT-qPCR analysis. The protein levels of viral E^rns^ (**G**), E1 (**I**), and E2 (**K**) and porcine IL-10 (**H**,**J**,**L**) at 12, 24, 36, and 48 hpt were measured by ELISA (* *p* < 0.05, ** *p* < 0.01, *** *p* < 0.001, **** *p* < 0.0001).

The previous experiment showed that CSFV up-regulated IL-10 expression in vivo and in vitro. To determine whether the CSFV glycoproteins regulate IL-10 expression, as an upstream target gene of the STAT1 pathway, the IL-10 mRNA levels were detected in PK-15 cells separately transfected with pCMV-E^rns-His^, pCMV-E1^-Myc^, and pCMV-E2^-Flag^ by RT-qPCR. IL-8, TGFβ1, TGFβ3, CCR2, CCR3, and IFNγ mRNA levels were also assessed. The mRNA levels of three glycoproteins were up-regulated in PK-15 cells (Figure 6A,C,E), while the mRNA expression of IL-10, TGFβ1, and IFNγ was up-regulated by ≥2.0-fold. Notably, the mRNA expression of IL-10 was significantly up-regulated by ≥4.0-fold at 36 hpt (Figure 6B,D,F). Moreover, IL-10 protein expression was measured in PK-15 cells transfected with pCMV-E^rns-His^, pCMV-E1^-Myc^, and pCMV-E2^-Flag^ by ELISA. E^rns^, E1, and E2 protein expression was up-regulated in vitro (Figure 6G,I,K), while the protein level of IL-10 was up-regulated (Figure 6H,J,L).

These results indicate that the CSFV glycoproteins (E^rns^, E1, and E2) directly up-regulated the expression of IL-10.

### 3.7. IL-10 Up-Regulated STAT1 Expression In Vitro

**Figure 7:**
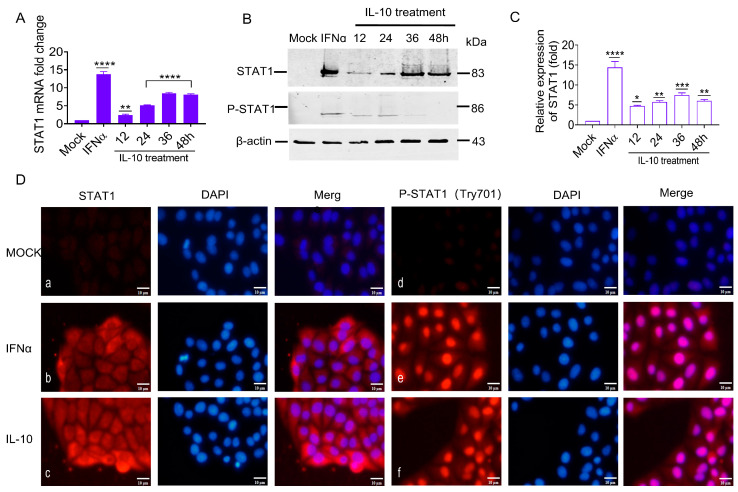
IL-10 up-regulated STAT1 expression in vitro. (**A**,**B**) Following porcine IL-10 (100 IU/mL) and porcine IFNα (100 IU/mL) treatment, the transcription and protein levels of STAT1 at 12, 24, 36, and 48 h were measured by RT-qPCR and WB analysis. (**C**) The relative expression values of STAT1 protein were quantified using Image J software. (**D**) Subcellular localization of STAT1 in PK-15 cells separately treated with IL-10 (100 IU/mL) and IFNα (100 IU/mL). The expression of STAT1 (a–c) and P-STAT1 (d–f) proteins were evaluated at 48 h using anti-STAT1 and anti-P-STAT1 antibodies by IFA analysis (* *p* < 0.05, ** *p* < 0.01, *** *p* < 0.001, **** *p* < 0.0001). Original images of the western blots can be found at Supplementary Materials.

The previous experiments confirmed that the IL-10 gene was up-regulated in vivo and in vitro by CSFV glycoproteins. The aim was to ascertain if IL-10 was the key upstream gene of the STAT1 pathway based on CSFV infection in an alternative pathway independent of IFNα/β. In this study, porcine IL-10 was used to incubate PK-15 cells, and STAT1 transcriptional and protein levels were evaluated by RT-qPCR and WB analyses. The STAT1 transcriptional levels were significantly up-regulated by 2.3-, 5.1-, 8.3-, and 8.1-fold (Figure 7A), while the STAT1 protein levels were up-regulated by 4.7-, 5.7-, 7.4-, and 5.9-fold at 12, 24, 36, and 48 h (Figure 7B,C). These results suggest that IL-10 up-regulated STAT1 expression in vitro.

Our findings suggest that the STAT1 protein was produced in the cytoplasm and transferred to the nucleus in response to the CSFV E^rns^, E1, and E2. It remained unclear whether increasing the amount of IL-10 would also lead to the phosphorylation of STAT1 and subsequently affect its cellular localization. Therefore, PK-15 cells were incubated with IL-10 at 100 IU/mL to determine the effect on the activation, phosphorylation, and cellular localization of STAT1. The results of IFA showed that the specific red fluorescence of STAT1 and P-STAT1 was only slightly detectable in mock cells (Figure 7D: a and d). Contrarily, STAT1 was observed in both the cytoplasm and nucleus of IFNα-treated PK-15 cells, while P-STAT1 was found only in the nucleus (Figure 7D: b and e). In IL-10-treated PK-15 cells, STAT1 was found in both cytoplasm and nucleus, while P-STAT1 was only detected in the nucleus (Figure 7D: c and f), indicating that IL-10 is functionally similar to IFNα, which promoting STAT1 phosphorylation and nuclear translocation.

These results indicate that STAT1 mRNA and protein expression, phosphorylation, and nuclear translocation were positively correlated with IL-10 induction in vitro, implying that IL-10 is an upstream target of the STAT1 pathway and can directly up-regulate STAT1 expression in vitro.

### 3.8. Knockdown of IL-10 Down-Regulated STAT1 Expression

To clarify the relationship between IL-10 and STAT1, we wanted to determine if STAT1 expression in either transfected or CSFV-infected PK-15 cells could still be induced with IL-10 expression knocked-down via siRNA. Changes to the mRNA and protein levels of STAT1 were assessed in PK-15 cells that were initially treated with 100 nM IL-10 siRNA for 6 h and then transfected with pCMV-E^rns-His^, pCMV-E1^-Myc^, or pCMV-E2^-Flag^.

**Figure 8:**
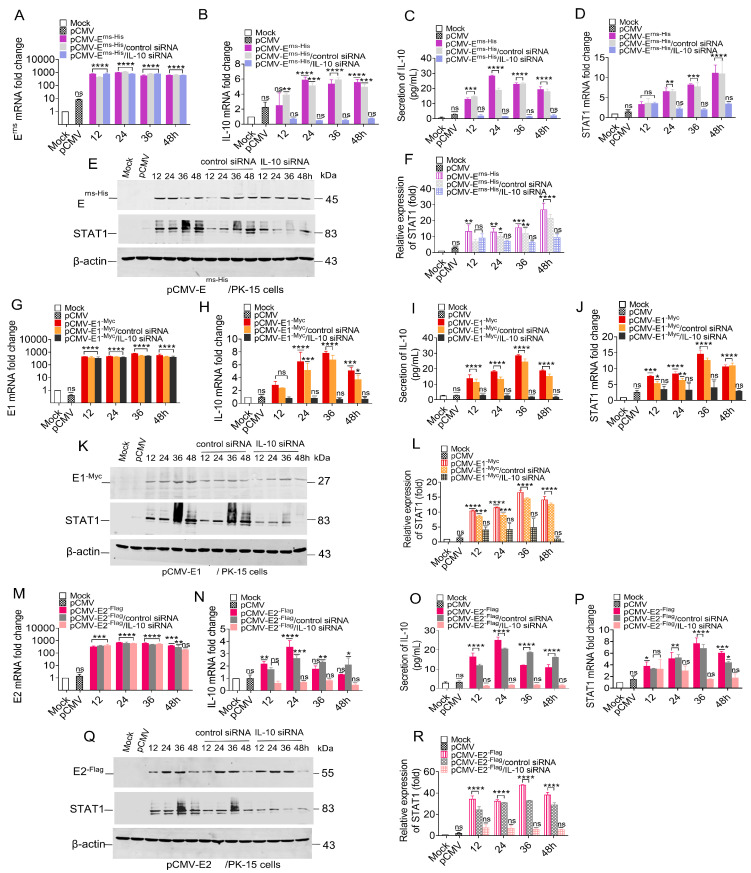
Knockdown of IL-10 down-regulated STAT1 expression. The transcription and protein levels of E^rns^, E1, E2, IL-10, and STAT1 have been detected by RT-qPCR, ELISA, and WB analysis in PK-15 cells temporarily transfected with IL-10 siRNA or control siRNA (100 nM) for 6 h, and then transfected with the pCMV-E^rns-His^ (**A**–**F**), pCMV-E1^-Myc^ (**G**–**L**), or pCMV-E2^-Flag^ (**M**–**R**) vectors, respectively, underlying the transfection of the empty vector pCMV (1.0 μg) as a negative control at 12, 24, 36, and 48 hpt. The mRNA levels of E^rns^, E1, and E2 (**A**,**G**,**M**), IL-10 (**B**,**H**,**N**), and STAT1 (**D**,**J**,**P**) are displayed by RT-qPCR analysis. The protein levels of IL-10 (**C**,**I**,**O**) and STAT1 (**E**,**K**,**Q**) were detected by ELISA and WB analysis. (**F**,**L**,**R**) The relative expression values of the STAT1 protein were quantified using Image J software (* *p* < 0.05, ** *p* < 0.01, *** *p* < 0.001, **** *p* < 0.0001). Original images of the western blots can be found at Supplementary Materials.

PK-15 cells were temporarily transfected with the pCMV-E^rns-His^, resulting in a relative increase in the mRNA levels of E^rns^, IL-10, and STAT1 (Figure 8A–F). A temporary transfection was conducted in PK-15 cells using 100 nM IL-10 siRNA for a duration of 6 h, followed by the transfection of the pCMV-E^rns-His^ vector at a concentration of 1.0 μg. Consequently, both the mRNA and protein levels of IL-10 were significantly down-regulated to less than 2-fold (Figure 8B,C). Furthermore, the mRNA and protein levels of STAT1 were also markedly diminished (Figure 8D,F).

PK-15 cells were used to express E1 protein by transfecting with pCMV-E1^-Myc^, and the mRNA levels of E1, IL-10 and STAT1 were up-regulated (Figure 8G–L). A temporary transfection was conducted in PK-15 cells using 100 nM IL-10 siRNA for a duration of 6 h, followed by the transfection of the pCMV-E1^-Myc^ vector at a concentration of 1.0 μg. Consequently, both the mRNA and protein levels of IL-10 were significantly down-regulated to less than 2-fold (Figure 8H,I). Moreover, the mRNA and protein levels of STAT1 were also down-regulated (Figure 8J,L).

PK-15 cells were temporarily transfected with pCMV-E2^-Flag^, resulting in a relative increase in the mRNA levels of E2, IL-10, and STAT1 (Figure 8M–R). A temporary transfection was conducted in PK-15 cells using 100 nM IL-10 siRNA for a duration of 6 h, followed by the transfection of the pCMV-E2^-Flag^ vector at a concentration of 1.0 μg. The results show that the IL-10 mRNA and protein levels were significantly decreased by less than 2.0-fold (Figure 8N,O), and STAT1 mRNA and protein levels were also decreased (Figure 8P–R).

Collectively, these results indicate that all three CSFV glycoproteins (E^rns^, E1, and E2) mediated STAT1 expression via IL-10 and that the siRNA-induced knockout of IL-10 down-regulated STAT1 expression.

### 3.9. Identification of Downstream Targets of the STAT1 Pathway

To determine gene targets downstream of the now-altered STAT1 pathway associated with CSFV infection, we collected PBMCs from CSFV-infected piglets at 1, 3, 6, and 9 dpi and analyzed them via microarray to determine the fold changes in OAS1, MX1, and ISG20 expression levels. The results revealed dynamic changes in mRNA expression over the course of infection. Specifically, the OAS1 mRNA levels in PBMCs from CSFV-infected piglets exhibited fold changes of −4.6, −1.03, 5.97, and 2.27 at 1, 3, 6, and 9 dpi, respectively. Similarly, the MX1 mRNA expression showed fold changes of −1.09, 4.98, 3.96, and 1.9 at the corresponding time points. In contrast, the ISG20 mRNA expression demonstrated more pronounced changes, with fold changes of 2.36, 17.38, 12.03, and 5.07 at 1, 3, 6, and 9 dpi, respectively (Table 1).

**Figure 9:**
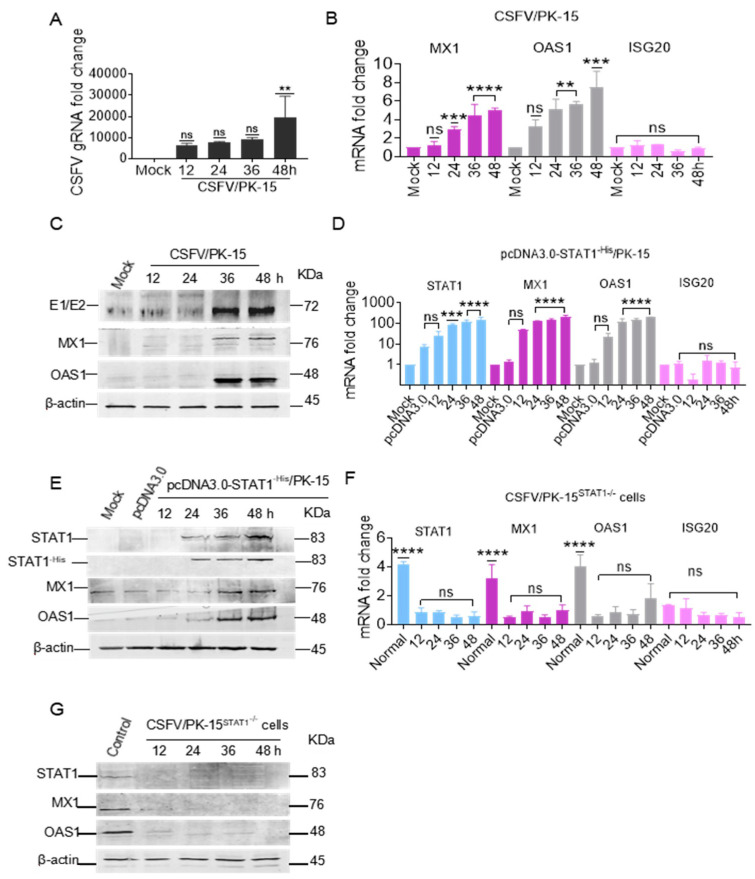
Identification of downstream targets of the STAT1 pathway. (**A**,**B**) The copy number of CSFV gRNA and the mRNA levels of porcine MX1, OAS1, and ISG20 were quantified through RT-qPCR analysis at 12, 24, 36, and 48 hpi in PK-15 cells infected with CSFV (MOI = 1). (**C**) The protein levels were assessed using Western blot (WB) analysis. (**D**) The mRNA levels of porcine STAT1, MX1, OAS1, and ISG20 were quantified using RT-qPCR analysis at 12, 24, 36, and 48 hpt in PK-15 cells transfected with the pcDNA3.0-STAT1^-His^ vector, and (**E**) protein levels were assessed through WB analysis. (**F**) The mRNA levels of porcine STAT1, MX1, OAS1, and ISG20 were quantified using RT-qPCR analysis at 12, 24, 36, and 48 hpi in CSFV-infected PK-15^STAT1-/-^ cells and (**G**) protein levels were measured by WB analysis (** *p* < 0.01, *** *p* < 0.001, **** *p* < 0.0001). Original images of the western blots can be found at Supplementary Materials.

The mRNA and protein levels of OAS1 and MX1 were elevated in PK-15 cells infected with CSFV, concomitant with an increase in the copy number of the CSFV genome (Figure 9A). Notably, there was no change to the mRNA levels of ISG20 (Figure 9B). The protein levels of MX1 and OAS1 were increased at 36 and 48 hpi, which is consistent with the expression of viral protein (Figure 9C), implying that the mRNA and protein levels of MX1 and OAS1, but not ISG20, were up-regulated during CSFV infection.

To assess the influence of STAT1 on downstream genes, the mRNA levels of OAS1, MX1, and ISG20 were measured in PK-15 cells transfected with pcDNA3.0-STAT1^-His^. The results showed that STAT1 mRNA levels were up-regulated by 83.7-, 123.4-, and 157.7-fold at 24, 36, and 48 hpt, respectively, while MX1 mRNA levels were up-regulated by 128.9-, 150.3-, and 207.1-fold, and OAS1 mRNA levels were up-regulated by 126.2-, 152.7-, and 200.6-fold. However, there was no change to the mRNA levels of ISG20 (Figure 9D). The protein levels of MX1 and OAS1 were consistent with the expression profiles of STAT1 in PK-15 cells transfected with pcDNA3.0-STAT1^-His^ (Figure 9E), revealing that STAT1 overexpression directly up-regulated the expression of the downstream targets MX1 and OAS1, but not ISG20.

To confirm the influence of STAT1 on the expression profiles of OAS1, MX1, and ISG20, PK-15^STAT1-/-^ cells were infected with CSFV and the mRNA levels of STAT1, MX1, OAS1, and ISG20 were evaluated. There were no obvious changes to the mRNA levels of STAT1, MX1, OAS1, and ISG20 in CSFV-infected PK-15^STAT1-/-^ cells (Figure 9F). Furthermore, the STAT1, MX1, and OAS1 proteins were not detected in CSFV-infected PK-15^STAT1-/-^ cells (Figure 9G). These results indicate that both MX1 and OAS1 are downstream targets of the STAT1 pathway during CSFV infection.

## 4. Discussion

The interferon (IFN)-mediated antiviral response represents a crucial pathway for host defense against viral infections. At least two pathways have been proposed to induce IFN by recognizing exogenous RNA. One such pathway is initiated by TLR3, which specifically responds to exogenous double-stranded RNA (dsRNA) that may be released during the lysis of infected cells [29]. The alternative pathway is initiated by RIG-I and MDA5; this activation occurs in response to the accumulation of intracellular dsRNA during viral genome replication [29]. Both TLR3 and RIG-I/MDA5 interact with various molecules to activate IRF3 and NF-κB, ultimately leading to the expression of IFNα/β.

CSFV is an important infectious agent in the swine industry. CSFV infection triggers the secretion of cytokines and the synthesis of antibodies [29]; this viral infection activates downstream signaling molecules, including MAVS, IRF3, and NF-κB, through the engagement of receptors such as RIG-I, MDA5, and TLR3 (with a primary focus on RIG-I). The resultant activation facilitates the production of various inflammatory cytokines, notably IL-1β, IL-6, IL-8, and TNF-α [26,28]. Notably, while CSFV infection can stimulate the production of IFN-III, its up-regulation both in vivo and in vitro remains limited. IFNα/β exerts antiviral activity by binding to the IFNAR1/IFNAR2 complex during viral infection [39,40]. However, viruses continue to evolve various defense strategies to combat the IFNα/β-produced immune response. Our study confirmed that CSFV infection inhibited IFNα/β expression (Figure 1).

The identification of the JAK-STAT pathway emerged from significant research into the mechanisms underlying cellular responses to IFN. STAT1 is a major component of the JAK-STAT pathway in response to IFNα/β. Upon stimulation by IFNα/β, STAT1 phosphorylates and transports into the nucleus to activate transcription of interferon-stimulated genes (ISGs) [41]. Since IFNα/β is responsible for STAT1 activation, if CSFV infection inhibits IFNα/β, CSFV infection should also inhibit STAT1 expression. However, our study found that CSFV infection up-regulated STAT1 expression, but inhibited IFNα/β expression, suggesting that STAT1 up-regulation might be an effective strategy for protection of the host cell through an alternative to the IFNα/β pathway during CSFV infection. A non-infectious UV-treated CSFV induced STAT1 expression, demonstrating that STAT1 expression might be related to the outer structural proteins. Our study confirmed that CSFV up-regulated STAT1 expression and facilitated the nuclear transport of STAT1 via E^rns^, E1, and E2 individually (Figure 2, Figure 3 and Figure 4). Additional experiments will be needed to answer two questions: (1) why all the CSFV envelope proteins E^rns^, Et, and E2 have the same function of activating STAT1 expression and promoting nuclear translocation, and (2) whether the three proteins have a synergistic effect of STAT1 up-regulation. The E1/E2 and Erns/E2 heterodimers were found to exist on the surface of the virion to synergistically activate host immune response [42,43]. There are reasons to believe that E^rns^, E1, and E2 might synergistically promote STAT1 expression.

Besides IFNα/β, various factors have been found to activate STAT1, including interleukins (IL-6), growth factors (epidermal growth factor and platelet-derived growth factor), and cytokines (IFNγ, TGFβ1, and TNF) [44]. Our study confirmed that IL-10 was up-regulated in the PBMCs of piglets in addition to PK-15 cells in response to CSFV infection (Figure 5). The E^rns^, E1, and E2 separately induced IL-10 up-regulation and the subsequent up-regulation, phosphorylation, and nuclear translocation of STAT1 in PK-15 cells (Figure 6 and Figure 7). Silencing of IL-10 gene with siRNA down-regulated STAT1 expression (Figure 8). These results confirm that IL-10 can directly up-regulate STAT1 expression during CSFV infection. Although the mechanism underlying IL-10-induced STAT1 activity during CSFV infection remains unclear, a previous study found that JAK1 and JAK3/tyrosine kinase 2 are rapidly recruited to the STAT1 receptor and activated via IL-10 in anti-inflammatory responses [45]. Activated JAK catalyzes tyrosine phosphorylation of the STAT1 receptor by binding to the SH2 site, thereby forming a dimer, and promotes translocation into the nucleus, which directly affects the transcription of related genes [46]. IL-10 signaling via the JAK-STAT pathway is associated with host cell defense against potential pathogens [47].

Activated STAT1 induces the expression of a series of ISGs at various stages of viral replication to combat infection. Specifically, OAS1 synthesizes 2′-5′ oligoadenylate by recognizing the dsRNA of the virus. This process subsequently activates RNase L, which leads to the cleavage of viral RNA and ultimately inhibits viral protein synthesis [48]. Notably, both in vivo and in vitro studies have demonstrated that CSFV infection induces the expression of pOASL. Furthermore, pOASL interacts with pMDA5 in an RNase L-independent manner, enhancing IFN production and amplifying pMDA5-mediated antiviral signaling, thereby inhibiting CSFV replication [49]. MX1 functions at an early stage of the viral replication cycle, targeting viral nuclear proteins through its GTPase activity within the N-terminal GTPase domain [50]. Further investigation into the antiviral mechanism of MX1 revealed that the MX1 protein effectively inhibited the RdRp activity of CSFV NS5B [51]. Consistent with the previous reports, our results revealed that the mRNA levels of OAS1 and MX1 were increased in CSFV-infected PBMCs in vivo as well as PK-15 cells in vitro, while expression was down-regulated in CSFV-infected PK-15^STAT1-/-^ cells (Figure 9). These results indicate that both MX1 and OAS1 are downstream targets of the STAT1 pathway during CSFV infection.

**Figure 10:**
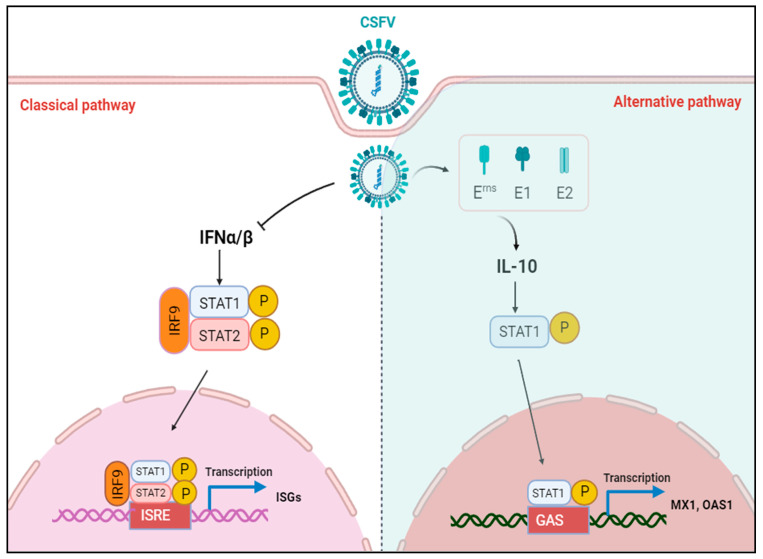
Substitution of the classical IFNα/β-STAT1 pathway with the IL-10-STAT1 pathway during CSFV infection. The type I IFN-mediated STAT1 pathway was replaced with the alternative IL-10-STAT1 pathway during CSFV infection via the viral envelope glycoproteins E^rns^, E1, and E2. The classical pathway: IFNα/β-p-STAT1/p-STAT2-IRF9-ISGs (**left** panel). The alternative pathway: IL-10-p-STAT1-MX1/OAS1 (**right** panel).

Overall, our study findings provide hitherto undocumented evidence of IL-10-induced STAT1 activation, independent of IFNα/β, mediated by the CSFV E^rns^, E1, and E2 proteins (Figure 10).

## 5. Conclusions

CSF is a major infectious disease that profoundly affects the swine industry. The widespread use of the rabbit-attenuated vaccine for swine fever has significantly altered the epidemiological dynamics of CSF. This shift has transformed the disease from an acute, widespread pandemic to a more localized and regionally distributed phenomenon. As a result, CSF has become increasingly complex, characterized by latent infections, mild or atypical clinical presentations, persistent infections, and frequent co-infections with multiple viral strains. Among the current challenges in the prevention and control of CSF, one of the most critical is effectively managing the issue of persistent infection with CSFV. To address this challenge, a deeper understanding of the interactions between CSFV infection and the host immune response is crucial.

Current research on IL-10-mediated JAK-STAT signaling pathways primarily emphasizes their role in antimicrobial defense mechanisms against potential pathogens. For instance, *Lactobacillus rhamnosus*—a commensal microbe present in the placental mucosa—has been shown to activate the IL-10 and JAK-STAT signaling pathways [52]. Furthermore, both IL-10 and STAT3 have been found to play a crucial role in maintaining intestinal tolerance and homeostasis during the progression of chronic enterocolitis in genetically deficient mice [53].

However, little is currently known about how IL-10 activates STAT1 during viral infections. Our study reveals that IL-10 serves as an alternative pathway to IFNα/β, directly upregulating STAT1 expression in the context of CSFV infection. This upregulation of STAT1 subsequently activates MX1 and OAS1, leading to the induction of antiviral effects (Figure 10).

Currently, understanding how pathogens modulate JAK-STAT signaling through IL-10-dependent or -independent pathways remains a compelling area of ongoing research.

## Figures and Tables

**Table 1:** Fold changes to mRNA levels of PBMCs of piglets infected with CSFV.

Gene	GenBank Accession No.	Fold Change to mRNA Levels of PBMCs of Piglets Infected with CSFV			
		1 dpi	3 dpi	6 dpi	9 dpi
*IFNα*	M28623	−1.16	−1.21	−1.19	−1.10
*IFNβ*	S41178	1.38	1.14	1.07	1.16
*STAT1*	NM213769	1.26	1.44	1.81	2.07
*OAS1*	NM214303.2	−4.6	−1.03	5.97	2.27
*MX1*	DQ095779.1	−1.09	4.98	3.96	1.9
*ISG20*	AY702647.2	2.36	17.38	12.03	5.07

## Data Availability

The data that support the findings of microarray are openly available at https://www.ncbi.nlm.nih.gov/geo/query/acc.cgi?acc=GSE276190 (accessed on 5 January 2024). The other data are contained within the article.

## Supplementary Materials

The following supporting information can be downloaded at: https://www.mdpi.com/article/10.3390/biom15020200/s1, Table S1: Primers used for detection of cytokines by RT-qPCR. Original images for Figure 1, Figure 2, Figure 3 and Figure 4, Figure 7, Figure 8 and Figure 9.
