# Supplementary figures and images for: Classical Swine Fever Virus Envelope Glycoproteins Erns, E1, and E2 Activate IL-10-STAT1-MX1/OAS1 Antiviral Pathway via Replacing Classical IFNα/β
Source: Biomolecules. 2025 Jan 31;15(2):200. doi: 10.3390/biom15020200 (PMC11853677; doi:10.3390/biom15020200)

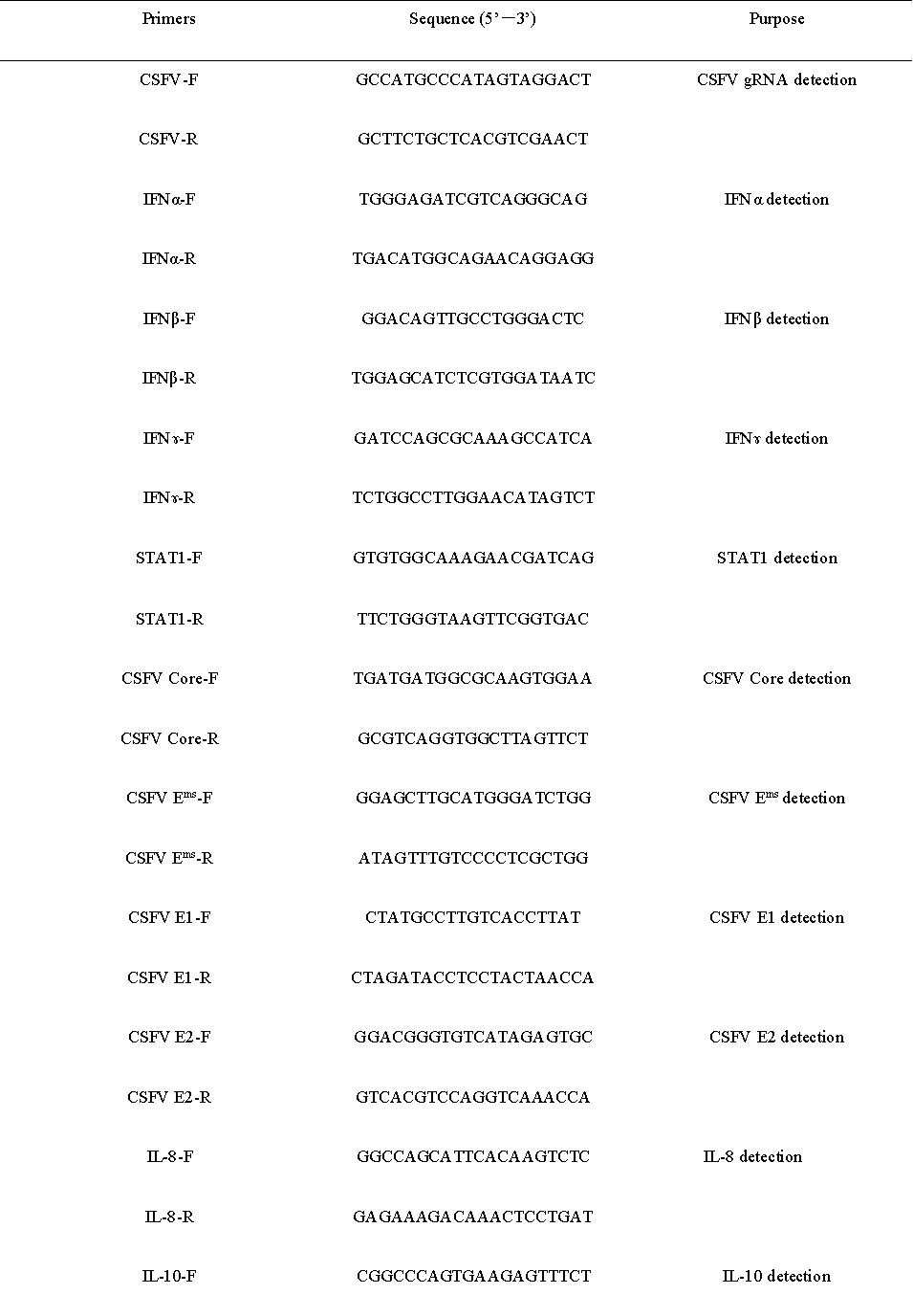

Supplement: Supplementary file 1 [file biomolecules-15-00200-s001.zip › S1 Table.png]

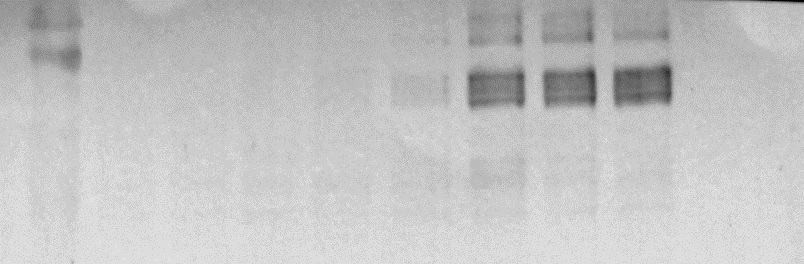

Supplement: Supplementary file 1 [file biomolecules-15-00200-s001.zip › WB raw data/Fig1B E1 E2.png]

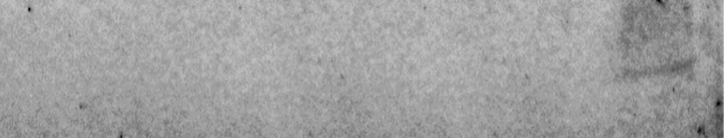

Supplement: Supplementary file 1 [file biomolecules-15-00200-s001.zip › WB raw data/Fig1B IFNα .png]

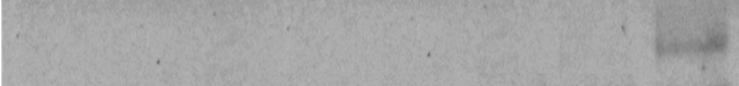

Supplement: Supplementary file 1 [file biomolecules-15-00200-s001.zip › WB raw data/Fig1B IFNβ.png]

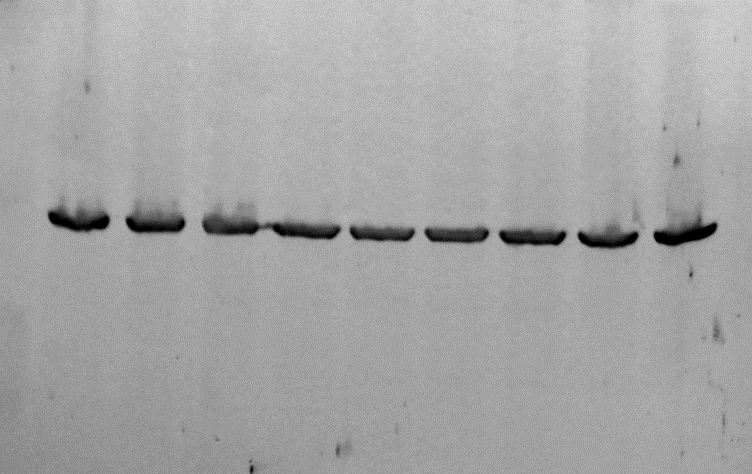

Supplement: Supplementary file 1 [file biomolecules-15-00200-s001.zip › WB raw data/Fig1B β-actin.png]

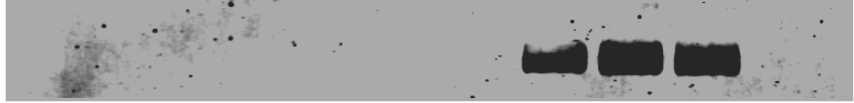

Supplement: Supplementary file 1 [file biomolecules-15-00200-s001.zip › WB raw data/Fig1E E1 E2.png]

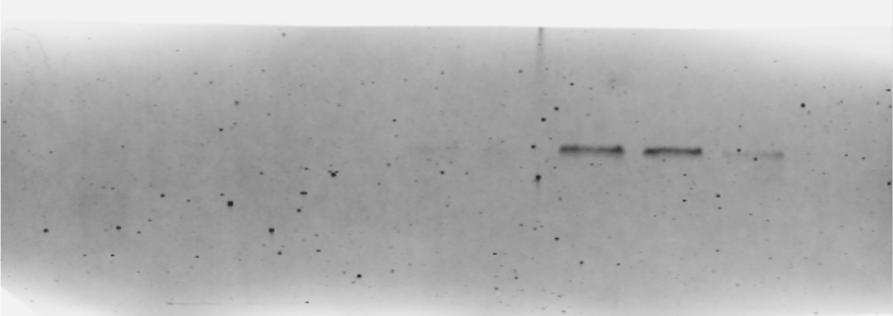

Supplement: Supplementary file 1 [file biomolecules-15-00200-s001.zip › WB raw data/Fig1E P-STAT1 .png]

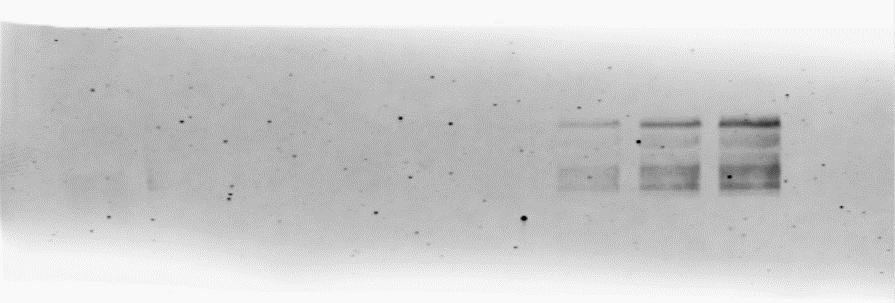

Supplement: Supplementary file 1 [file biomolecules-15-00200-s001.zip › WB raw data/Fig1E STAT1 .png]

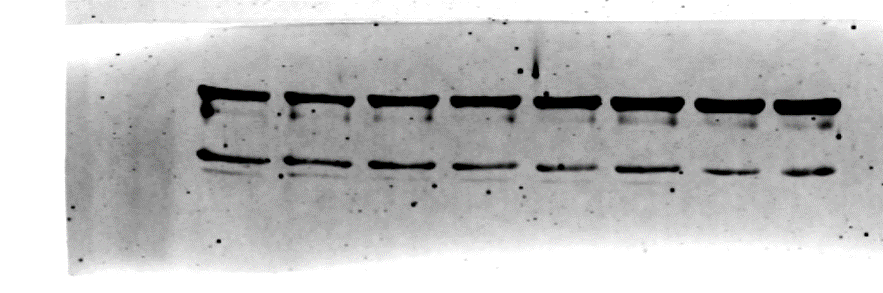

Supplement: Supplementary file 1 [file biomolecules-15-00200-s001.zip › WB raw data/Fig1E β-actin.png]

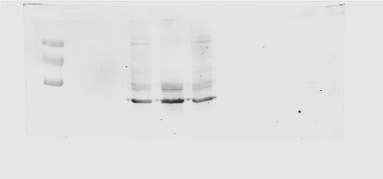

Supplement: Supplementary file 1 [file biomolecules-15-00200-s001.zip › WB raw data/Fig2D E2 .png]

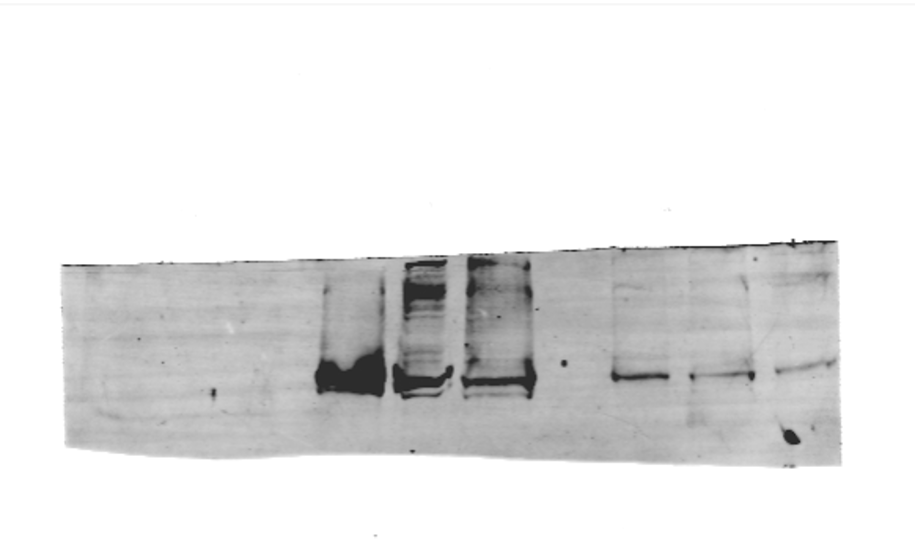

Supplement: Supplementary file 1 [file biomolecules-15-00200-s001.zip › WB raw data/Fig2D STAT1 .png]

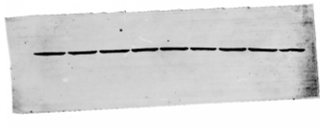

Supplement: Supplementary file 1 [file biomolecules-15-00200-s001.zip › WB raw data/Fig2D β-actin.png]

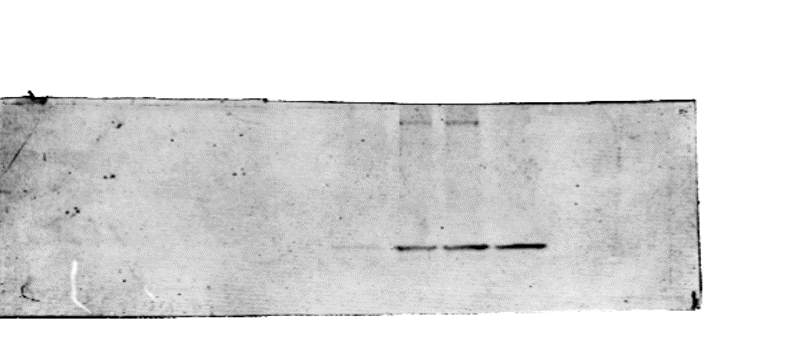

Supplement: Supplementary file 1 [file biomolecules-15-00200-s001.zip › WB raw data/Fig3C Core-HA.png]

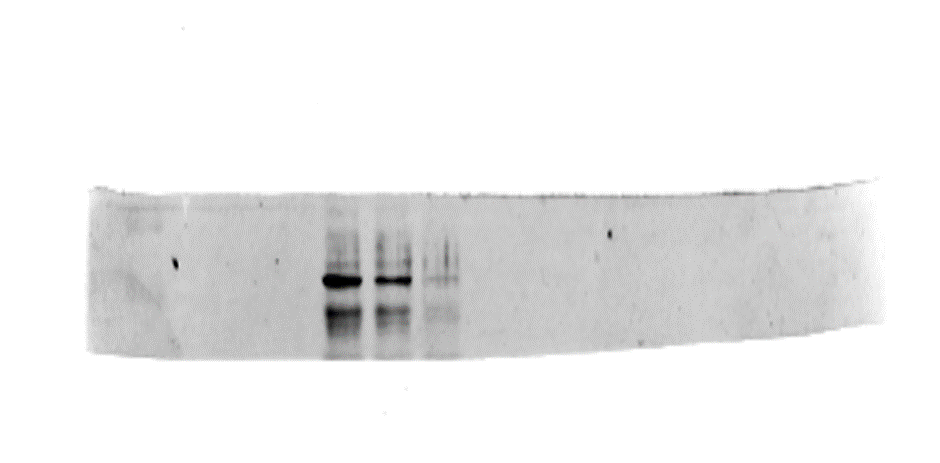

Supplement: Supplementary file 1 [file biomolecules-15-00200-s001.zip › WB raw data/Fig3C E2.png]

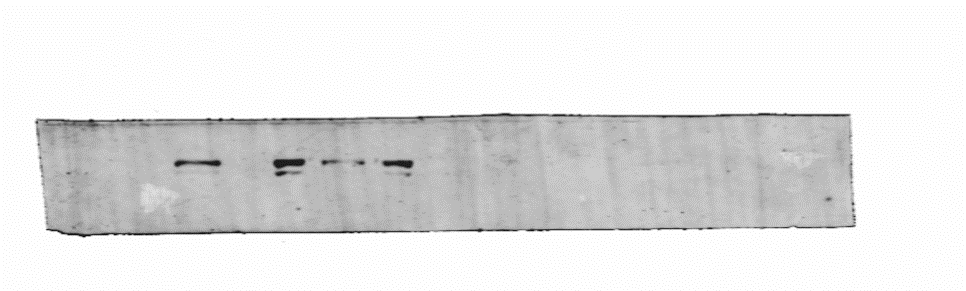

Supplement: Supplementary file 1 [file biomolecules-15-00200-s001.zip › WB raw data/Fig3C STAT1.png]

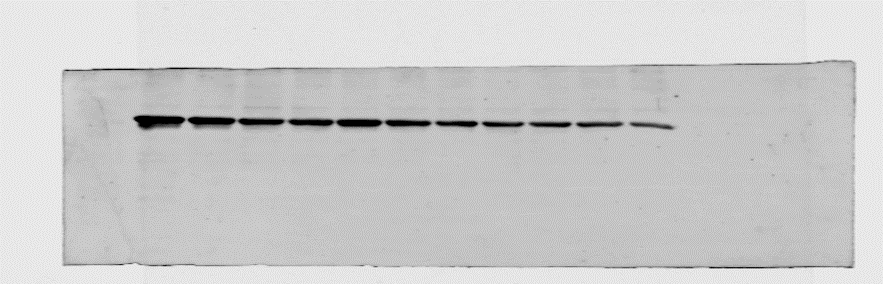

Supplement: Supplementary file 1 [file biomolecules-15-00200-s001.zip › WB raw data/Fig3C β-actin.png]

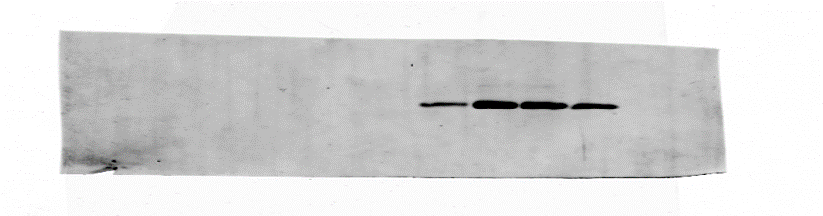

Supplement: Supplementary file 1 [file biomolecules-15-00200-s001.zip › WB raw data/Fig3G E-rns-His.png]

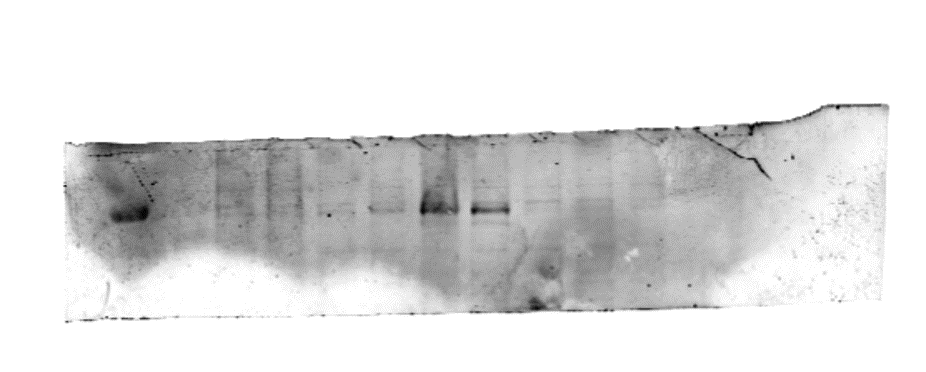

Supplement: Supplementary file 1 [file biomolecules-15-00200-s001.zip › WB raw data/Fig3G E2.png]

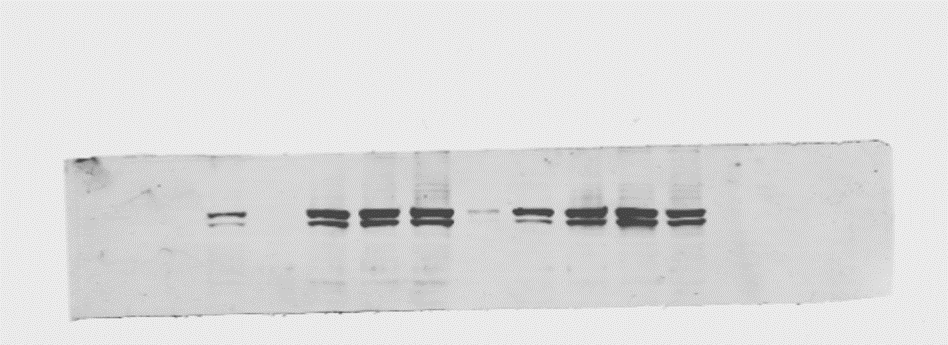

Supplement: Supplementary file 1 [file biomolecules-15-00200-s001.zip › WB raw data/Fig3G STAT1.png]

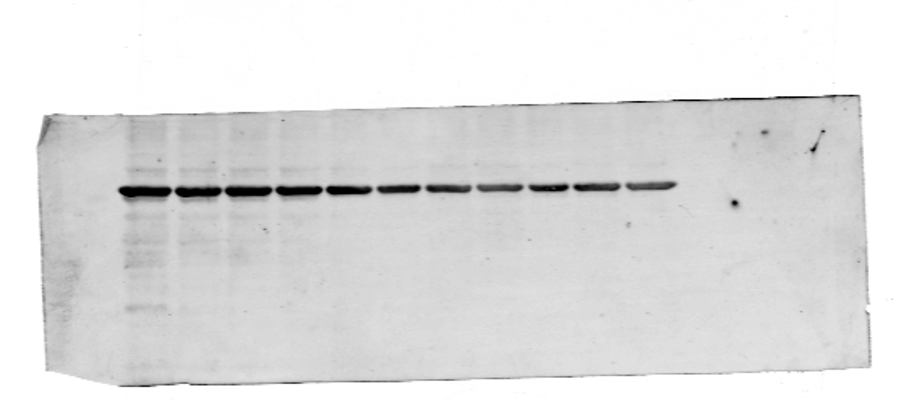

Supplement: Supplementary file 1 [file biomolecules-15-00200-s001.zip › WB raw data/Fig3G β-actin.png]

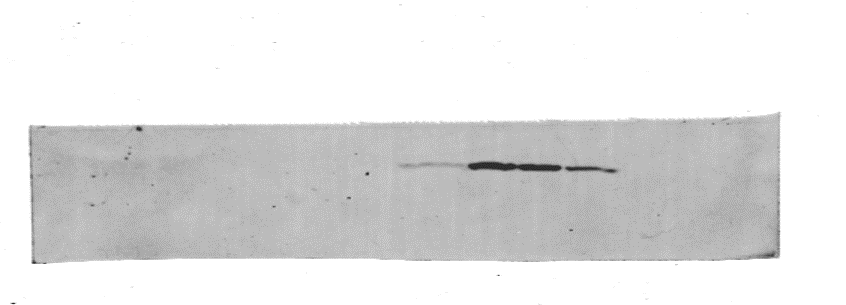

Supplement: Supplementary file 1 [file biomolecules-15-00200-s001.zip › WB raw data/Fig3K E1-Myc.png]

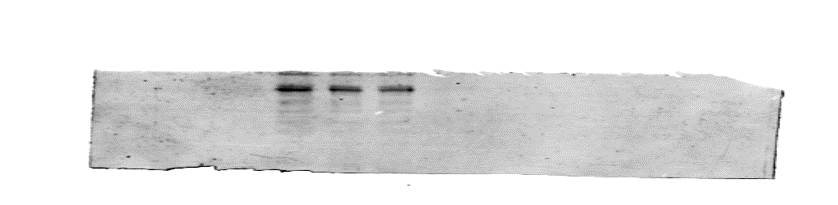

Supplement: Supplementary file 1 [file biomolecules-15-00200-s001.zip › WB raw data/Fig3K E2.png]

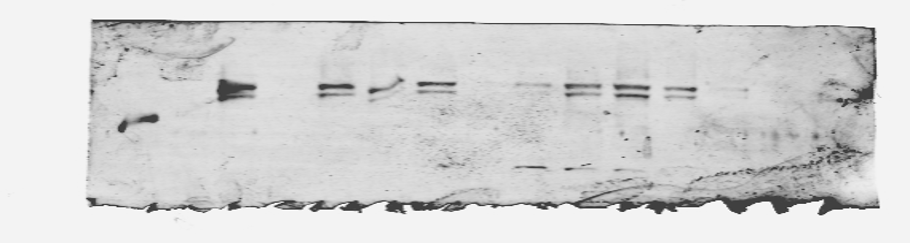

Supplement: Supplementary file 1 [file biomolecules-15-00200-s001.zip › WB raw data/Fig3K STAT1.png]

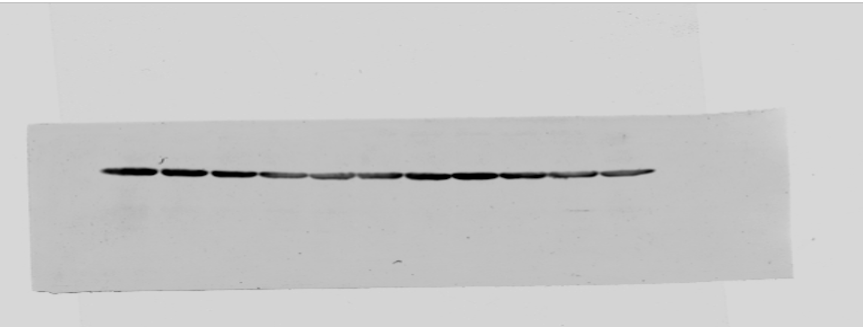

Supplement: Supplementary file 1 [file biomolecules-15-00200-s001.zip › WB raw data/Fig3K β-actin.png]

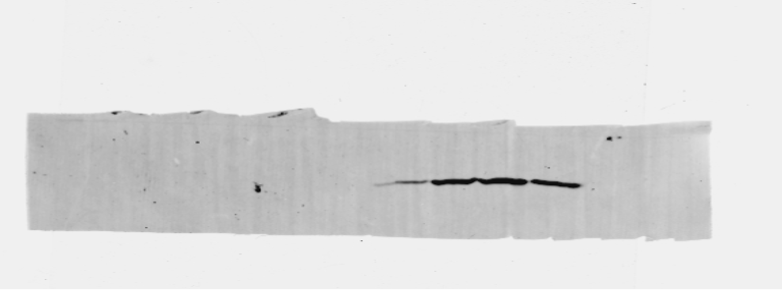

Supplement: Supplementary file 1 [file biomolecules-15-00200-s001.zip › WB raw data/Fig3O E2-Flag.png]

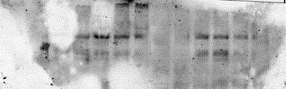

Supplement: Supplementary file 1 [file biomolecules-15-00200-s001.zip › WB raw data/Fig3O E2.png]

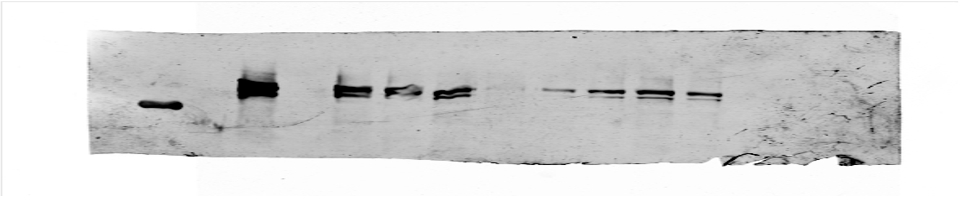

Supplement: Supplementary file 1 [file biomolecules-15-00200-s001.zip › WB raw data/Fig3O STAT1.png]

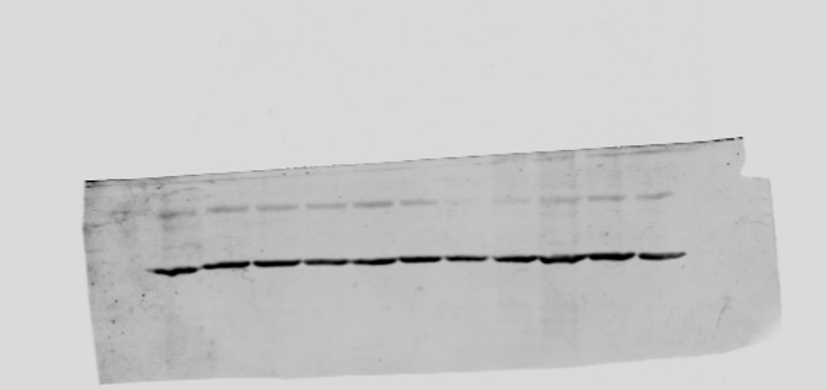

Supplement: Supplementary file 1 [file biomolecules-15-00200-s001.zip › WB raw data/Fig3O β-actin.png]

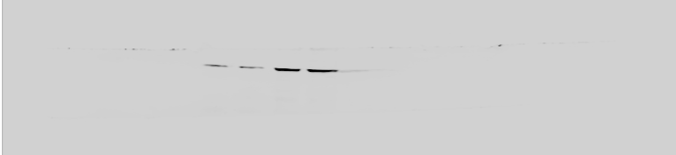

Supplement: Supplementary file 1 [file biomolecules-15-00200-s001.zip › WB raw data/Fig4A E-rns-His.png]

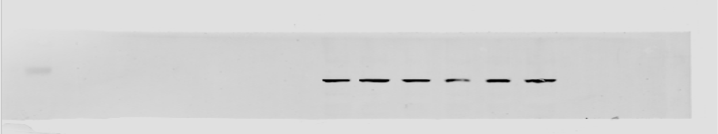

Supplement: Supplementary file 1 [file biomolecules-15-00200-s001.zip › WB raw data/Fig4A Lamin B1.png]

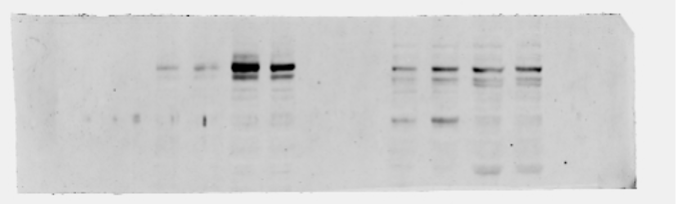

Supplement: Supplementary file 1 [file biomolecules-15-00200-s001.zip › WB raw data/Fig4A STAT1.png]

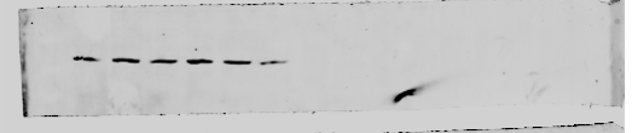

Supplement: Supplementary file 1 [file biomolecules-15-00200-s001.zip › WB raw data/Fig4A β-actin.png]

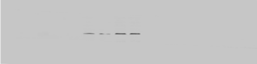

Supplement: Supplementary file 1 [file biomolecules-15-00200-s001.zip › WB raw data/Fig4C E1-Myc.png]

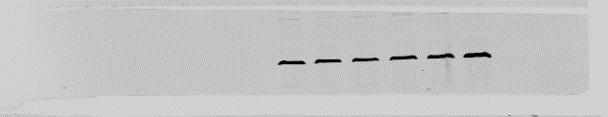

Supplement: Supplementary file 1 [file biomolecules-15-00200-s001.zip › WB raw data/Fig4C Lamin B1.png]

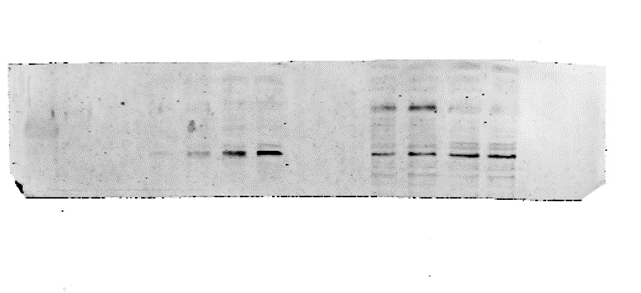

Supplement: Supplementary file 1 [file biomolecules-15-00200-s001.zip › WB raw data/Fig4C STAT1.png]

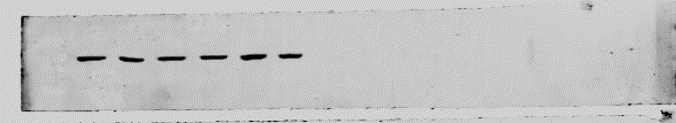

Supplement: Supplementary file 1 [file biomolecules-15-00200-s001.zip › WB raw data/Fig4C β-actin.png]

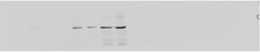

Supplement: Supplementary file 1 [file biomolecules-15-00200-s001.zip › WB raw data/Fig4E E2-Flag.png]

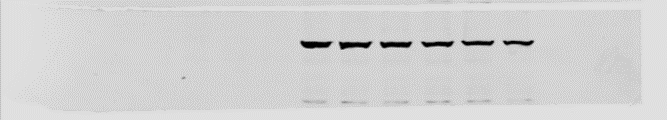

Supplement: Supplementary file 1 [file biomolecules-15-00200-s001.zip › WB raw data/Fig4E Lamin B1.png]

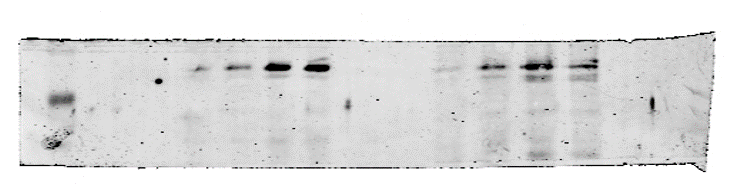

Supplement: Supplementary file 1 [file biomolecules-15-00200-s001.zip › WB raw data/Fig4E STAT1.png]

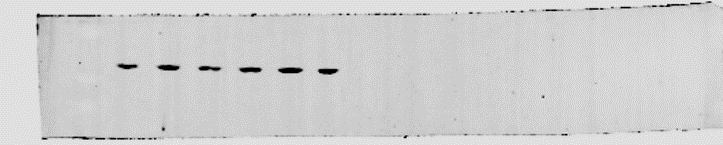

Supplement: Supplementary file 1 [file biomolecules-15-00200-s001.zip › WB raw data/Fig4E β-actin.png]

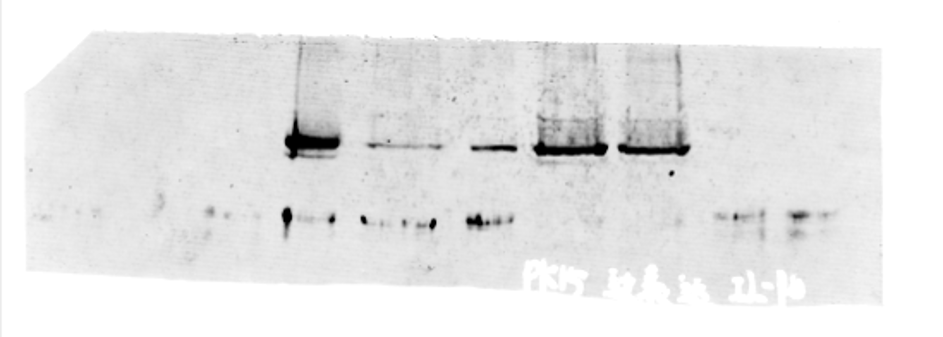

Supplement: Supplementary file 1 [file biomolecules-15-00200-s001.zip › WB raw data/Fig7B STAT.png]

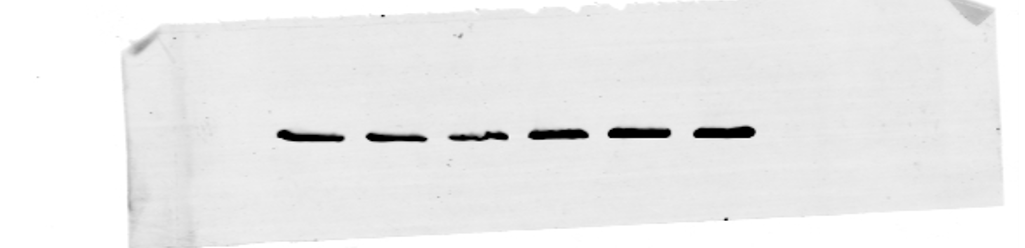

Supplement: Supplementary file 1 [file biomolecules-15-00200-s001.zip › WB raw data/Fig7B β-actin.png]

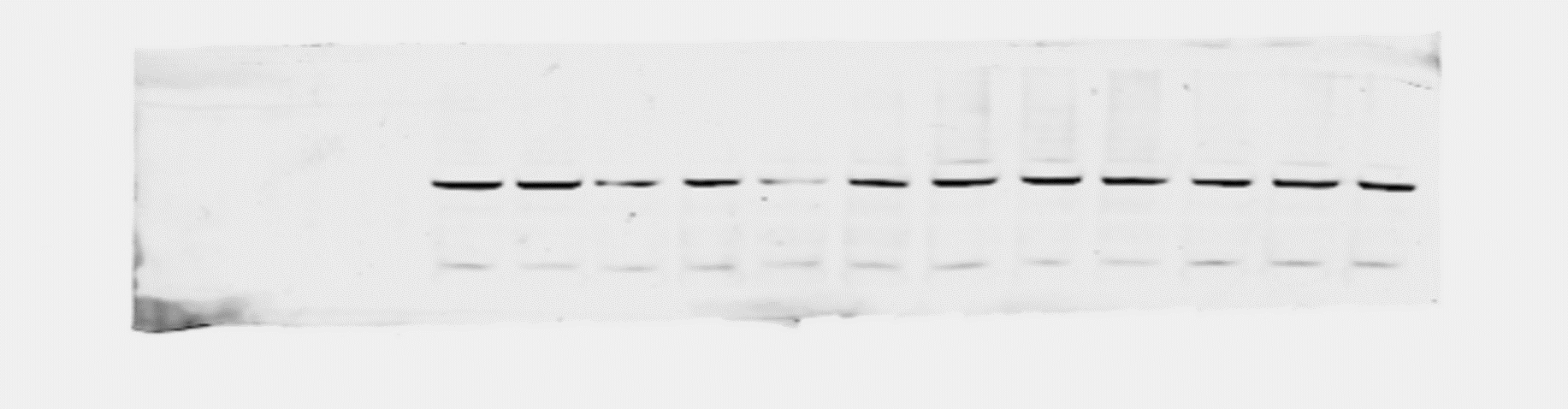

Supplement: Supplementary file 1 [file biomolecules-15-00200-s001.zip › WB raw data/Fig8E E-rns-His.png]

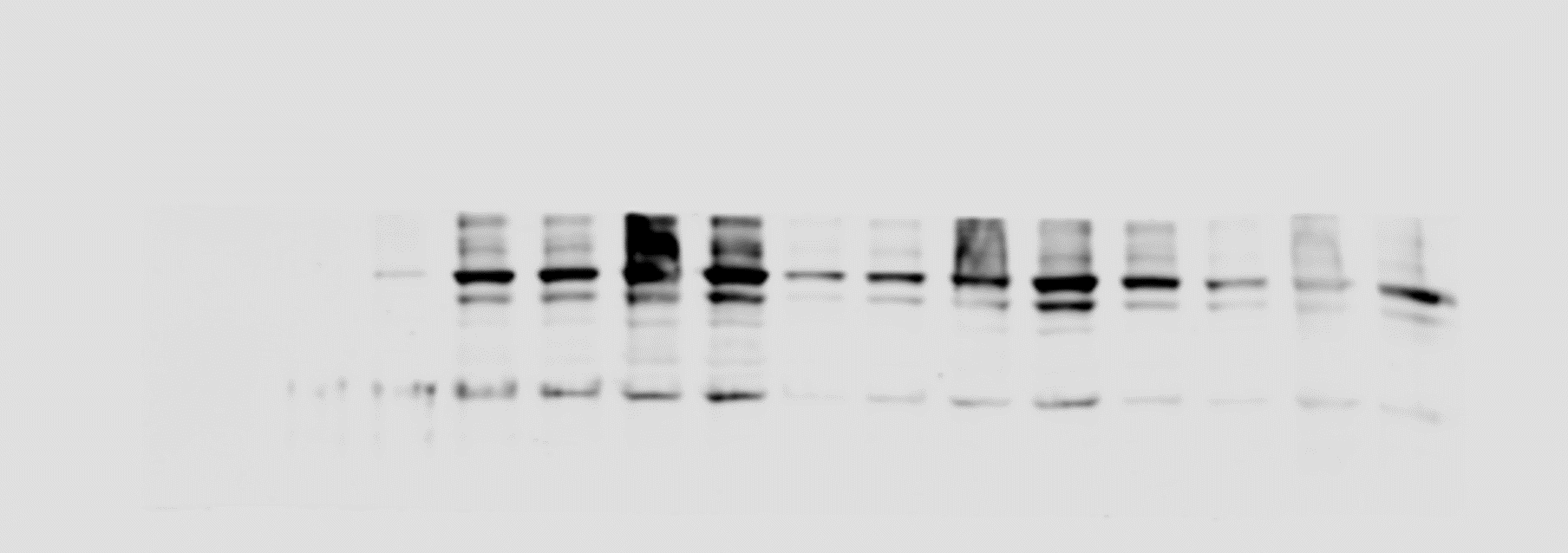

Supplement: Supplementary file 1 [file biomolecules-15-00200-s001.zip › WB raw data/Fig8E STAT1.png]

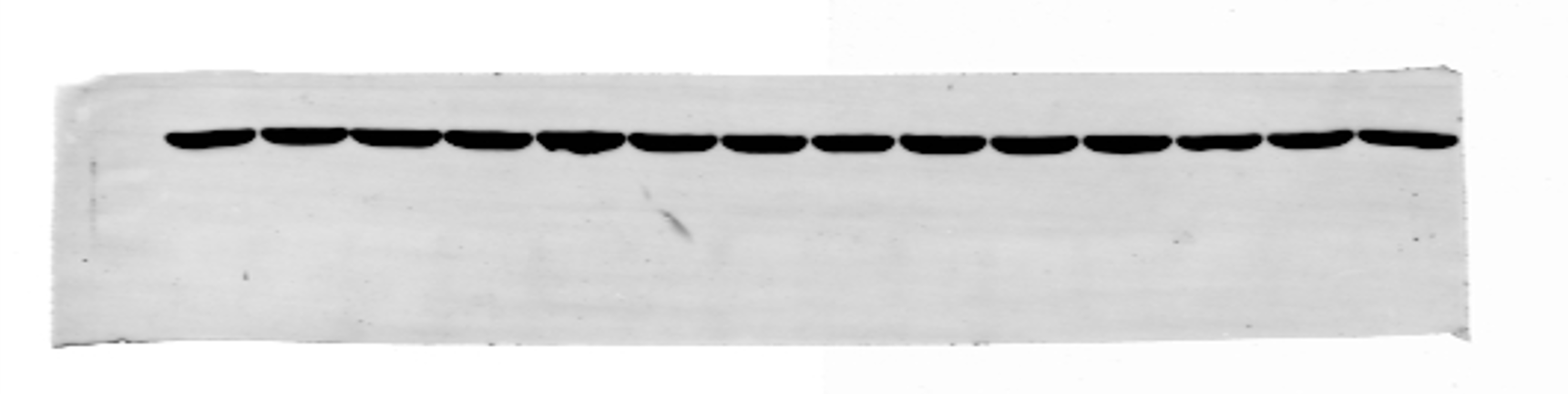

Supplement: Supplementary file 1 [file biomolecules-15-00200-s001.zip › WB raw data/Fig8E β-actin.png]

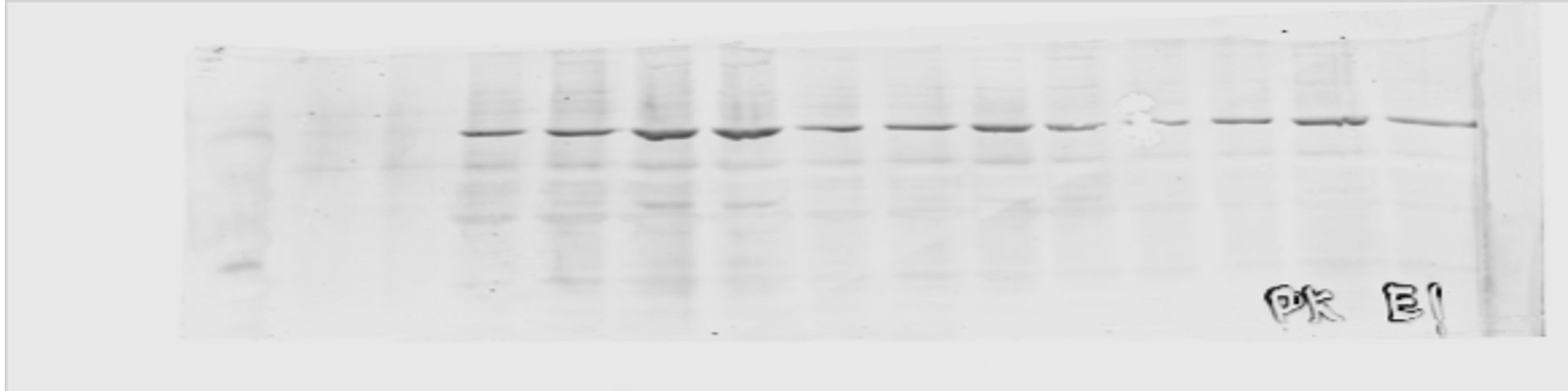

Supplement: Supplementary file 1 [file biomolecules-15-00200-s001.zip › WB raw data/Fig8K E1-Myc.png]

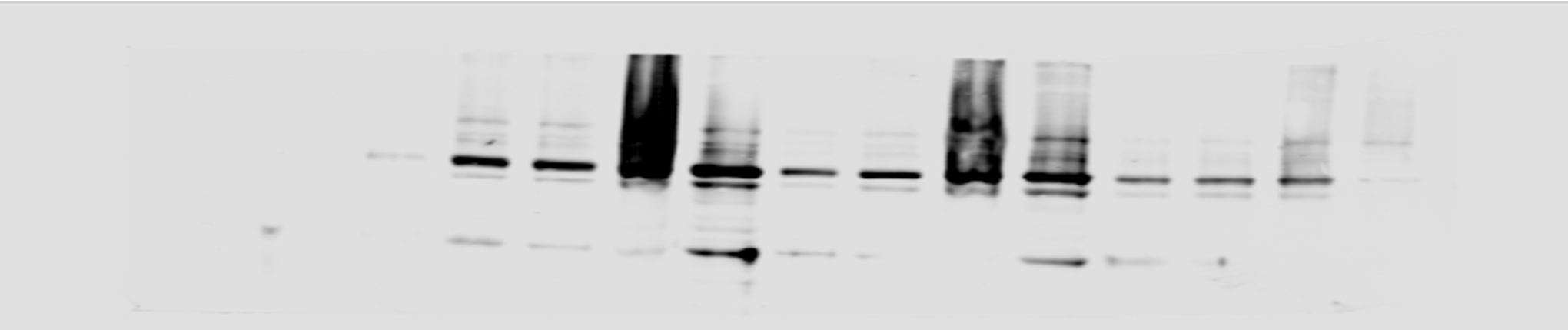

Supplement: Supplementary file 1 [file biomolecules-15-00200-s001.zip › WB raw data/Fig8K STAT1.png]

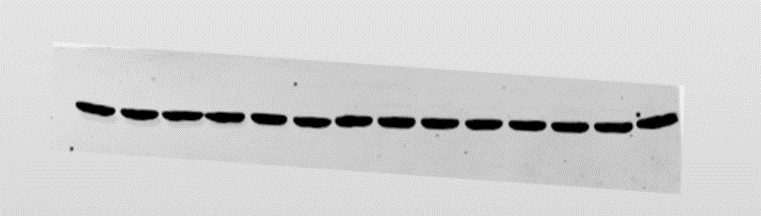

Supplement: Supplementary file 1 [file biomolecules-15-00200-s001.zip › WB raw data/Fig8K β-actin.png]

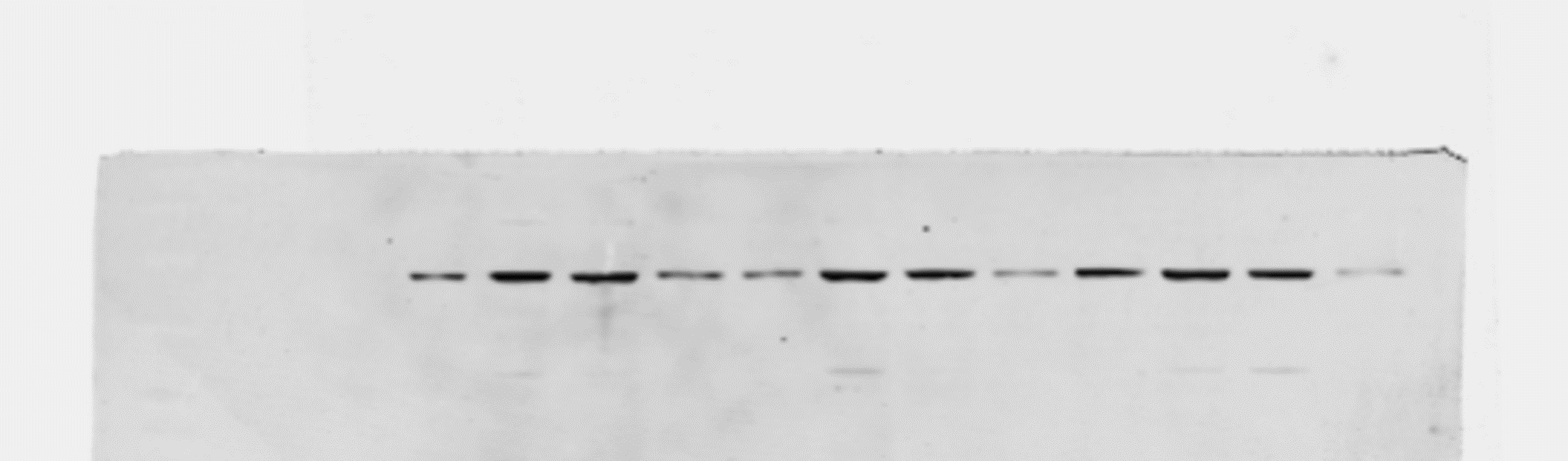

Supplement: Supplementary file 1 [file biomolecules-15-00200-s001.zip › WB raw data/Fig8Q E2-Flag.png]

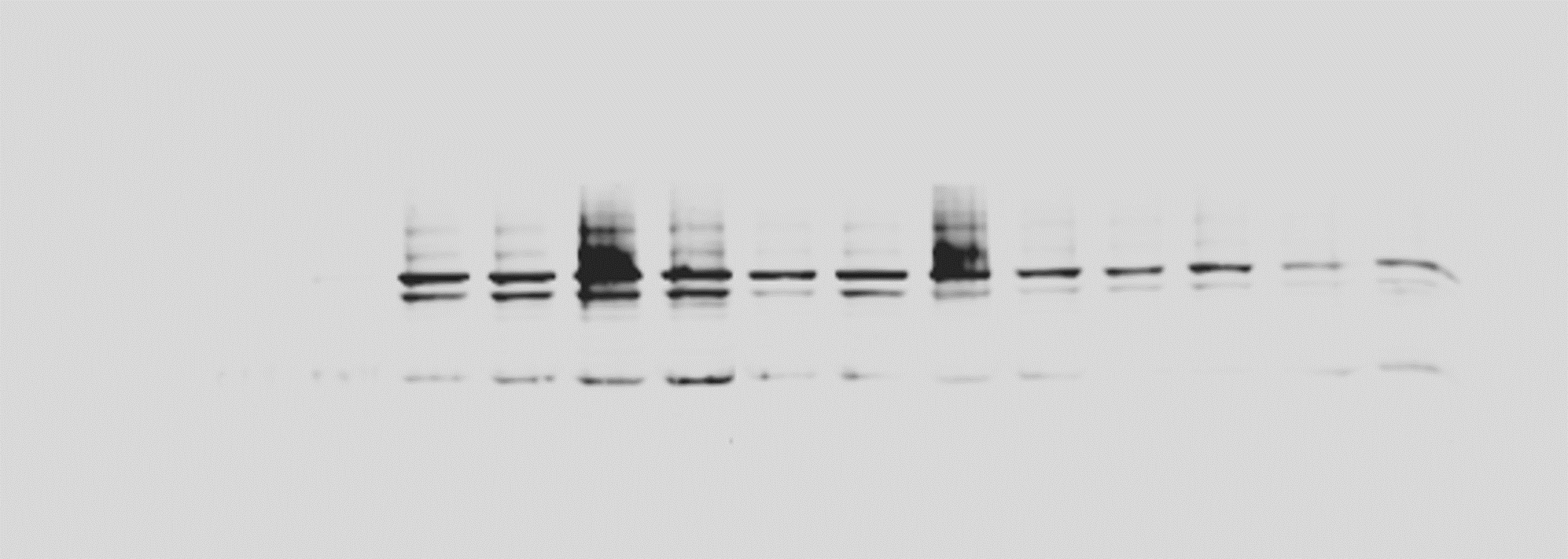

Supplement: Supplementary file 1 [file biomolecules-15-00200-s001.zip › WB raw data/Fig8Q STAT1.png]

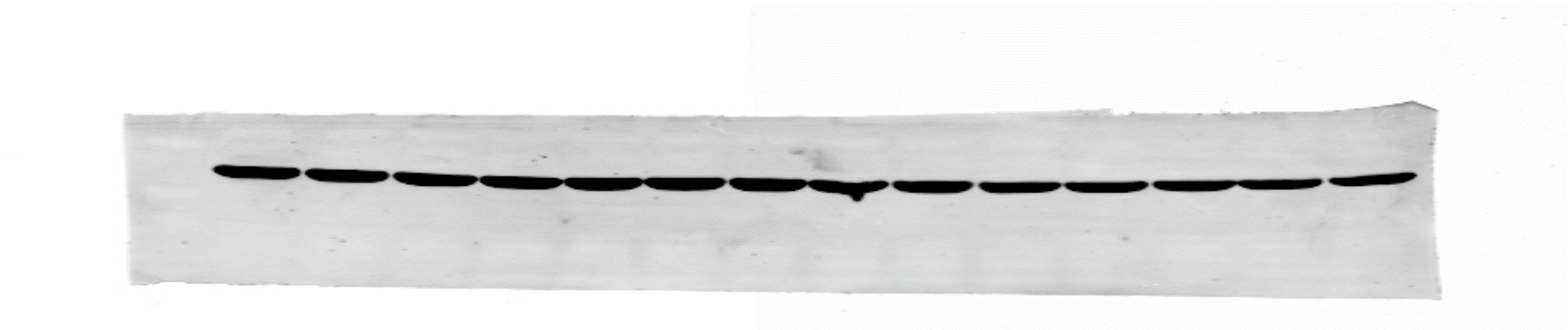

Supplement: Supplementary file 1 [file biomolecules-15-00200-s001.zip › WB raw data/Fig8Q β-actin.png]

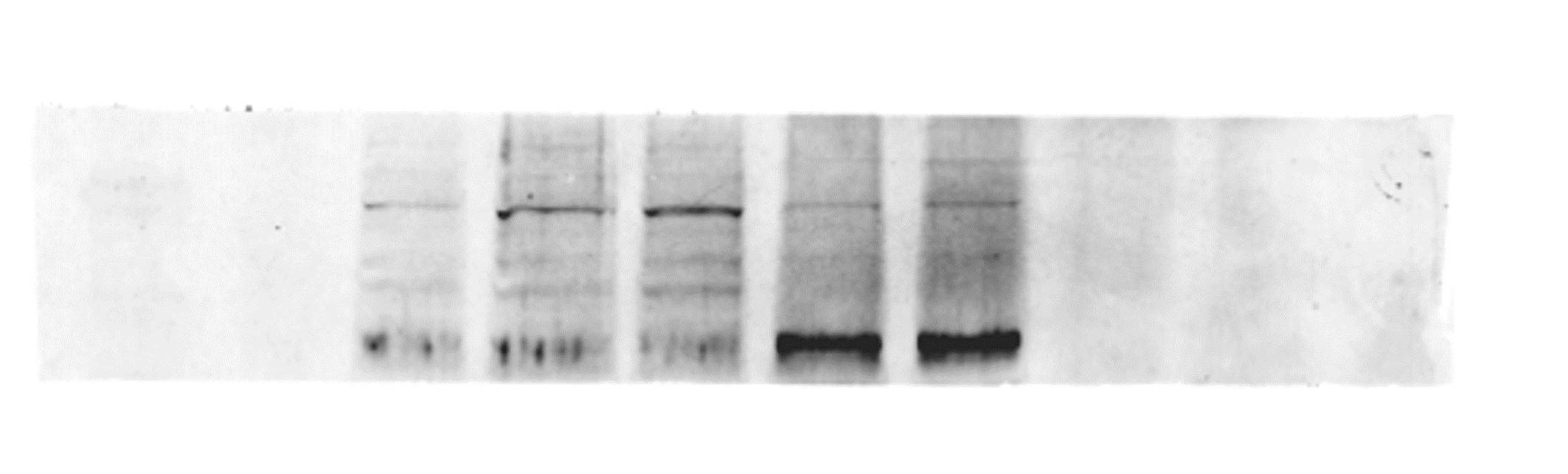

Supplement: Supplementary file 1 [file biomolecules-15-00200-s001.zip › WB raw data/Fig9C E1 E2.png]

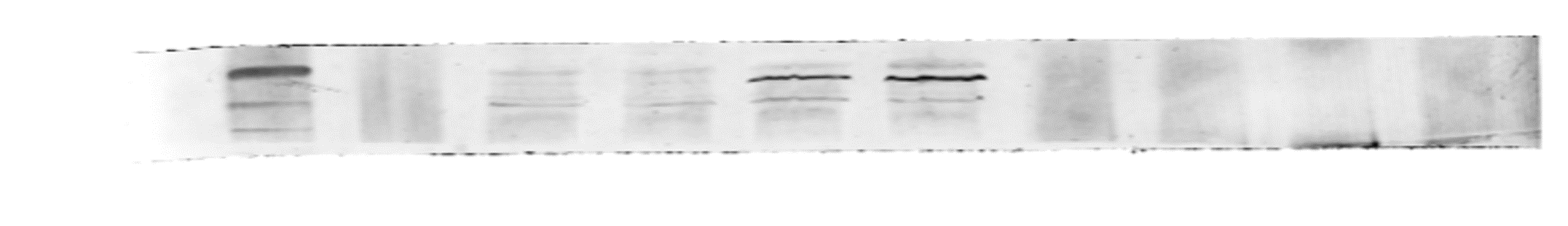

Supplement: Supplementary file 1 [file biomolecules-15-00200-s001.zip › WB raw data/Fig9C MX1.png]

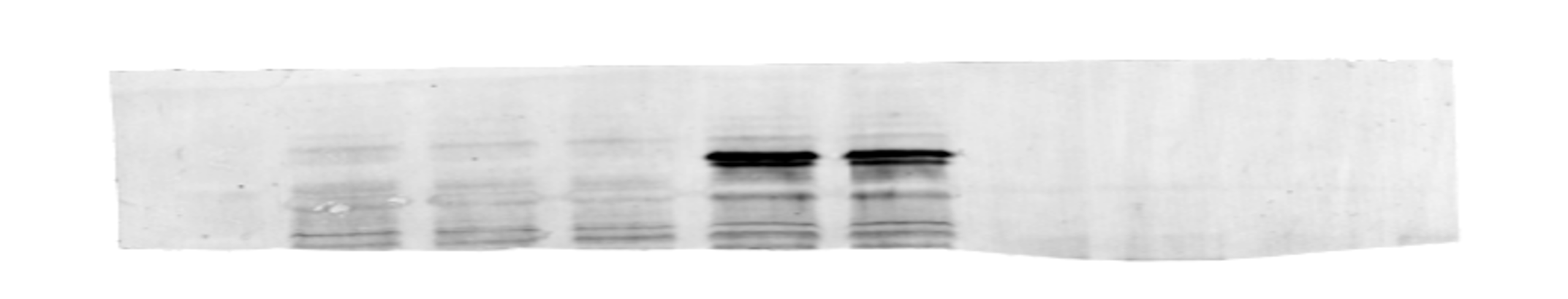

Supplement: Supplementary file 1 [file biomolecules-15-00200-s001.zip › WB raw data/Fig9C OAS1.png]

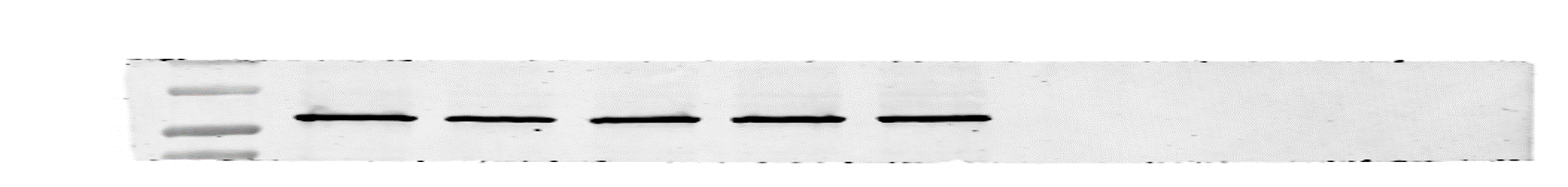

Supplement: Supplementary file 1 [file biomolecules-15-00200-s001.zip › WB raw data/Fig9C β-actin.png]

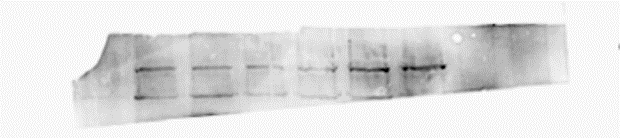

Supplement: Supplementary file 1 [file biomolecules-15-00200-s001.zip › WB raw data/Fig9E MX1.png]

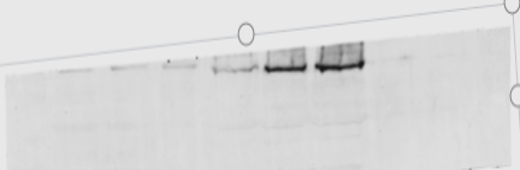

Supplement: Supplementary file 1 [file biomolecules-15-00200-s001.zip › WB raw data/Fig9E OAS1.png]

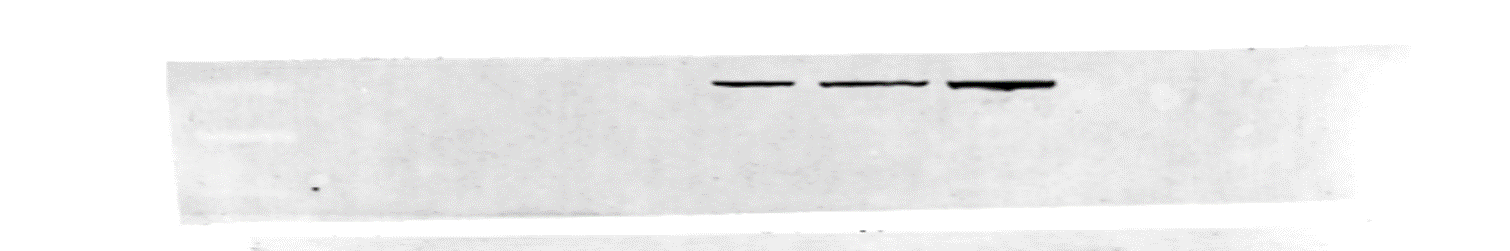

Supplement: Supplementary file 1 [file biomolecules-15-00200-s001.zip › WB raw data/Fig9E STAT1-His.png]

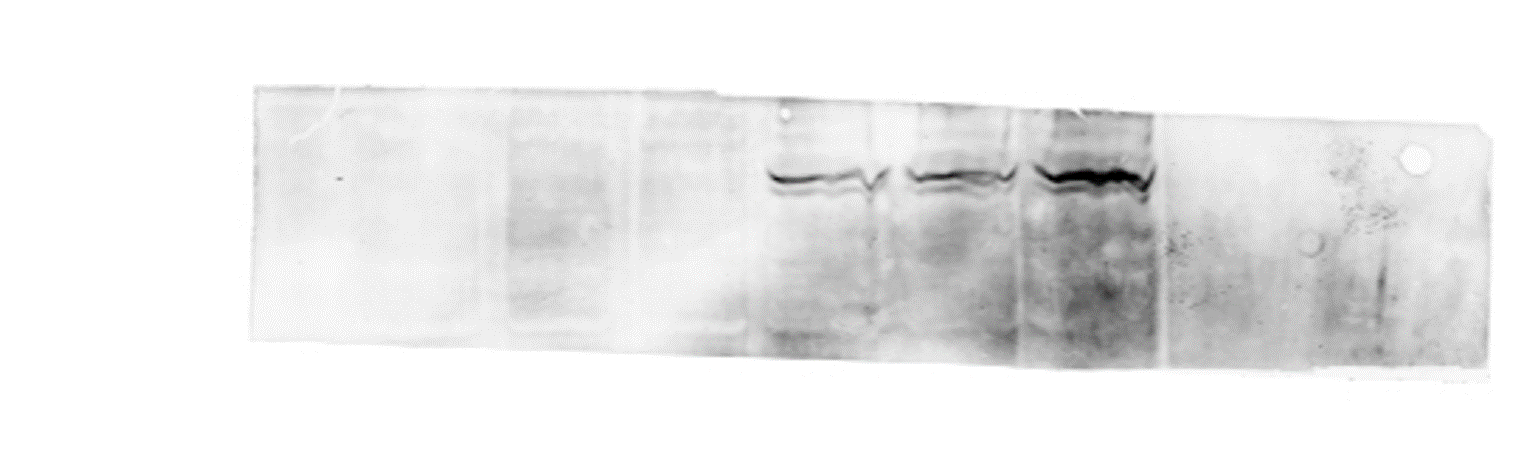

Supplement: Supplementary file 1 [file biomolecules-15-00200-s001.zip › WB raw data/Fig9E STAT1.png]

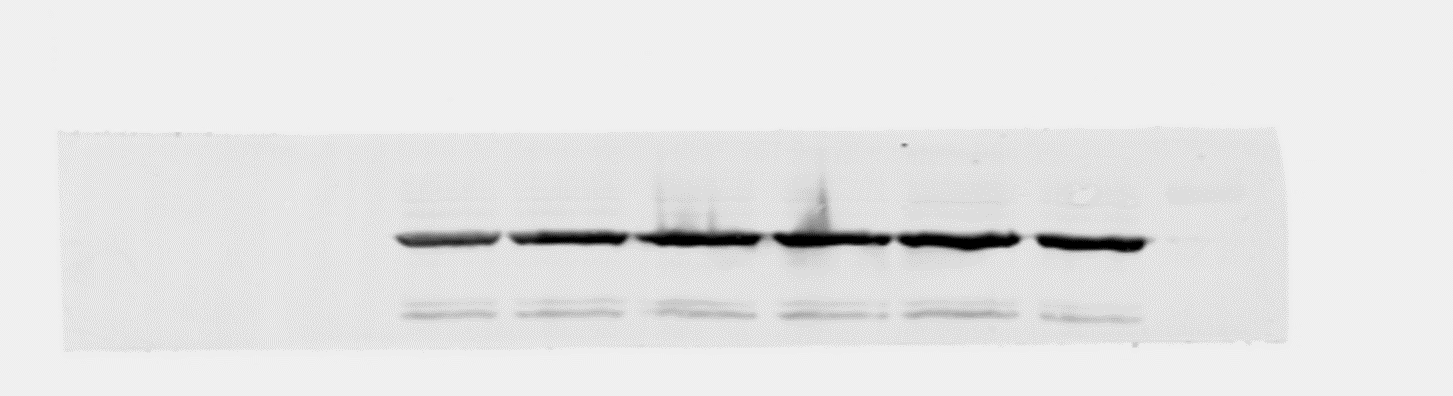

Supplement: Supplementary file 1 [file biomolecules-15-00200-s001.zip › WB raw data/Fig9E β-actin.png]

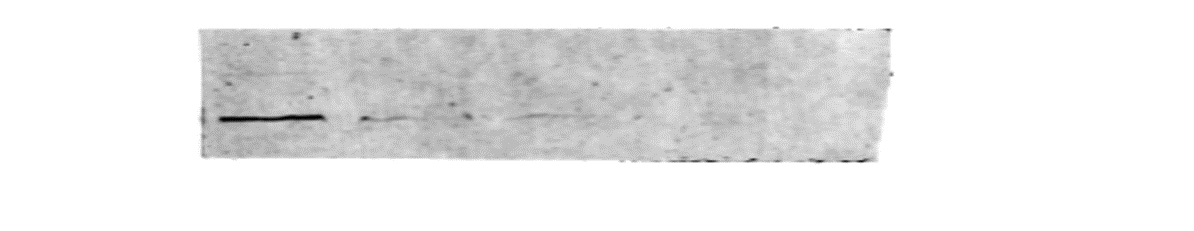

Supplement: Supplementary file 1 [file biomolecules-15-00200-s001.zip › WB raw data/Fig9G MX1.png]

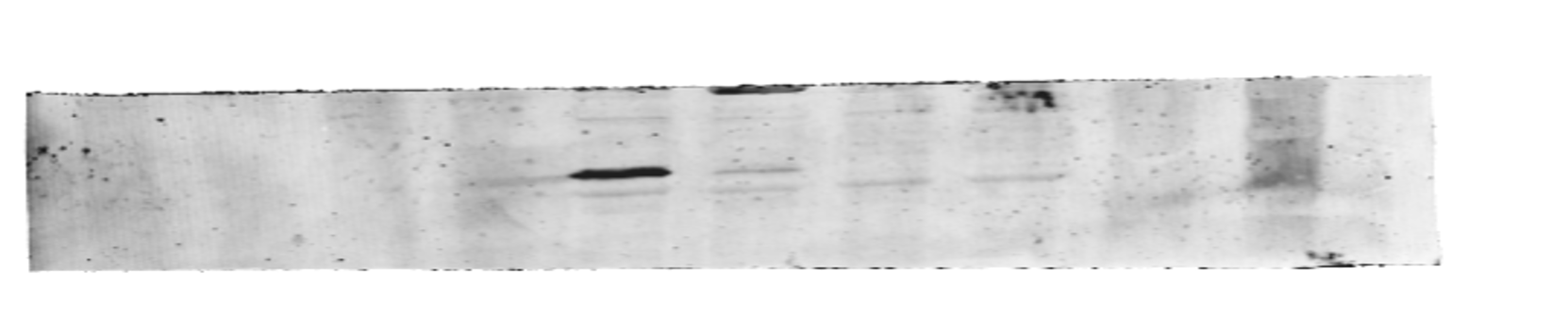

Supplement: Supplementary file 1 [file biomolecules-15-00200-s001.zip › WB raw data/Fig9G OAS1.png]

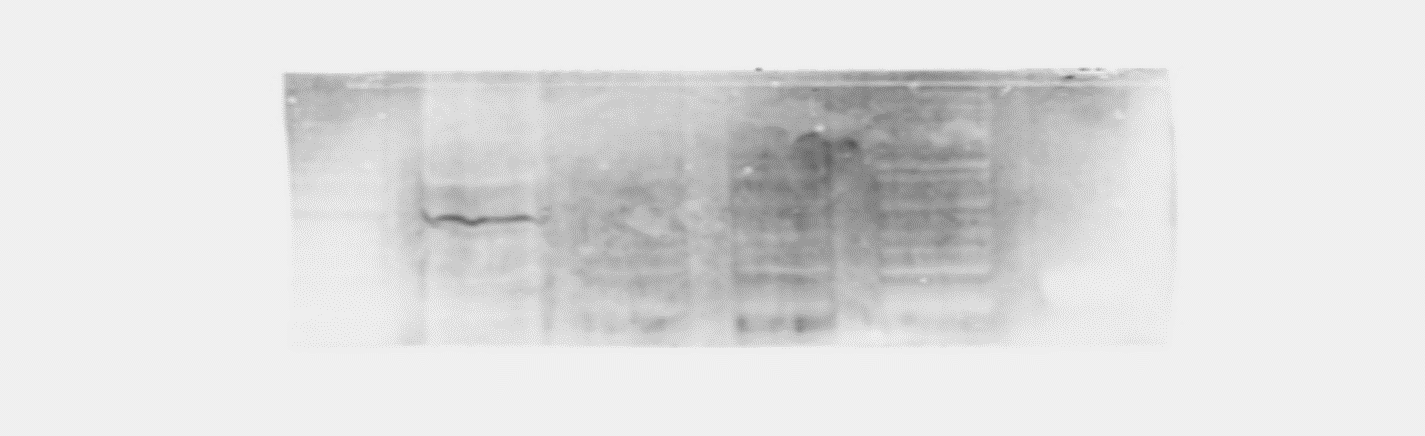

Supplement: Supplementary file 1 [file biomolecules-15-00200-s001.zip › WB raw data/Fig9G STAT1.png]

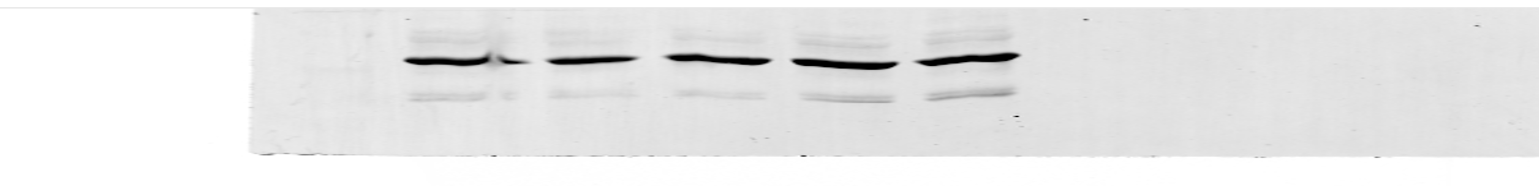

Supplement: Supplementary file 1 [file biomolecules-15-00200-s001.zip › WB raw data/Fig9G β-actin.png]
